# Towards rational design in electrochemical denitrification by analyzing pH dependence

**DOI:** 10.1093/nsr/nwae147

**Published:** 2024-04-16

**Authors:** Huan Li, Dong Luan, Jun Long, Xiaoyan Fu, Jianping Xiao

**Affiliations:** State Key Laboratory of Catalysis, Dalian Institute of Chemical Physics, Chinese Academy of Sciences, Dalian 116023, China; University of Chinese Academy of Sciences, Beijing 100049, China; State Key Laboratory of Catalysis, Dalian Institute of Chemical Physics, Chinese Academy of Sciences, Dalian 116023, China; State Key Laboratory of Catalysis, Dalian Institute of Chemical Physics, Chinese Academy of Sciences, Dalian 116023, China; State Key Laboratory of Catalysis, Dalian Institute of Chemical Physics, Chinese Academy of Sciences, Dalian 116023, China; State Key Laboratory of Catalysis, Dalian Institute of Chemical Physics, Chinese Academy of Sciences, Dalian 116023, China; University of Chinese Academy of Sciences, Beijing 100049, China

**Keywords:** electrocatalysis, denitrification, pH-dependence, nitric oxide reduction, reaction mechanism

## Abstract

A small fraction of NO*_x_* (<1%) always exists in CO_2_ feedstock (e.g. exhausted gas), which can significantly reduce the efficiency of CO_2_ electroreduction by ∼30%. Hence, electrochemical denitrification is the precondition of CO_2_ electroreduction. The pH effect is a key factor, and can be used to tune the selectivity between N_2_ and N_2_O production in electrochemical denitrification. However, there has been much controversy for many years about the origin of pH dependence in electrocatalysis. To this end, we present a new scheme to accurately model the pH dependence of the electrochemical mechanism. An extremely small pH variation from pH 12.7 to pH 14 can be accurately reproduced for N_2_O production. More importantly, the obviously different pH dependence of N_2_ production, compared to N_2_O, can be attributed to a cascade path. In other words, the N_2_ was produced from the secondary conversion of the as-produced N_2_O molecule (the major product), instead of the original reactant NO. This is further supported by more than 35 experiments over varying catalysts (Fe, Ni, Pd, Cu, Co, Pt and Ag), partial pressures (20%, 50% and 100%) and potentials (from −0.2 to 0.2 V vs. reversible hydrogen electrode). All in all, the insights herein overturn long-lasting views in the field of NO electroreduction and suggest that rational design should steer away from catalyst engineering toward reactor optimization.

## Teaser

The rational design for electrochemical denitrification, which is a necessary prerequisite for achieving high efficiency of CO_2_ electroreduction, should steer from catalyst engineering to reactor optimization.

## INTRODUCTION

**Figure 1.**
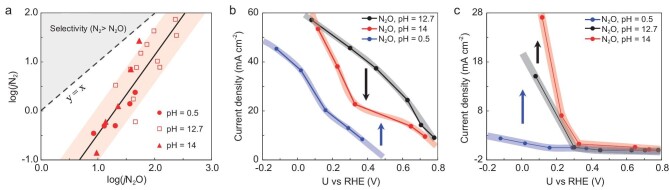
Overview of pH effects on electrochemical NO reduction (eNORR) over Pd [5]. (a) Strong correlation between experimental $\log ({{j}_{{{{\mathrm{N}}}_2}}})$ and $\log ({{j}_{{{{\mathrm{N}}}_2}{\mathrm{O}}}})$. Partial current densities of (b) N_2_O and (c) N_2_ production at pH 0.5, 12.7 and 14, respectively.

Nitric oxide (NO) removal is known to be a significant environmental issue [1,2]. Recently, its importance was stressed in CO_2_ electroreduction (eCO_2_RR). A trace of NO (∼0.8%) in feed can greatly lower the eCO_2_RR efficiency by ∼30% [3]. As a candidate to replace conventional selective catalytic reduction (SCR) [4], electrochemical denitrification (eNORR) can be driven by sustainable electricity and is a compatible route with eCO_2_RR in practical applications. An ideal target is to achieve high eNORR activity and N_2_ selectivity close to 100% at low overpotentials. It has been demonstrated that Pd is the most active transition metal, while its N_2_ selectivity is undesired [5,6]. As shown in Fig. 1, the N_2_ production is always less favorable compared to N_2_O production (below the diagonal line). More interestingly, the N_2_ selectivity is highly correlated with N_2_O production at varying pH values (Fig. 1a). It seems to be difficult to break the selectivity correlation in eNORR by pH effects. In other words, the N_2_ selectivity cannot be higher than 50% in eNORR.

However, there are obviously different behaviors for N_2_ and N_2_O production at varying pH, as shown in Fig. 1b and c. Generally speaking, alkaline electrolytes (pH 12.7) can enhance activity for both N_2_ and N_2_O production with respect to acid (pH 0.5) in eNORR. However, the activity of N_2_O production with increased pH is not monotonic. For instance, the current densities at pH 14 are lower than those at pH 12.7 at low overpotentials (>0.2 V vs. reversible hydrogen electrode (RHE)), while the activity (current density) can reverse at more negative potentials (Fig. 1b). In contrast to N_2_O production, the activity for N_2_ production at pH 14 is consistently higher than that at pH 12.7 for all potentials, as shown in Fig. 1c. Why are the pH effects so different between N_2_ and N_2_O production? It is seemingly contradictory, with the strong correlation between N_2_ and N_2_O production shown in Fig. 1a. One key to breaking the N_2_ selectivity limit of electrochemical denitrification is to understand the pH effects.

There is a lot of debate about the origin of pH dependence in electrocatalysis. To explain pH dependence, different assumptions have been made [7–17]. For instance, the theory of sequential proton-electron transfer (SPET) hypothesizes that proton transfer and electron transfer are decoupled and pH is the driving force for proton transfer [11,12,18,19]. In contrast, another assumption is that proton transfer is concerted with electron transfer; this is called a coupled proton-electron transfer (CPET), which is driven by the electrode potential vs. RHE. It has been widely used in electrocatalysis [20–27] and its pH dependence can be explained well by the electric field effect in thermodynamic analysis [10,28–30]. However, kinetic analysis for pH dependence remains challenging, to a large extent due to the difficulty in determining electrochemical barriers. Although there are some approaches for barrier calculations at constant potential [16,31–36], it is difficult to determine and accurately control the pH value in calculations. Besides, it is also difficult to model the pH dependence of barriers with an electric field, because the response of transition states (TSs) to a field cannot be calculated by those methods. Recently, we developed an approach [37] to control constant potential by keeping the electric field constant. It is called the electric field controlling constant potential (EFC-CP) method. This method can explicitly calculate the real structures of TSs at relative potentials (vs. RHE), where the response of TSs to the field is strictly considered. Nevertheless, modeling pH dependence requires the further determination of absolute potential vs. standard hydrogen electrode (SHE).

To this end, we present a new scheme based on the EFC-CP method to determine the SHE potential and electrochemical barriers at different pH conditions. A pH-dependent microkinetic model on Pd was employed to understand the considerably different pH effects on N_2_O and N_2_ production. According to the comparison between experiments and simulations, we found that N_2_O production is determined by two energetic factors, namely, NOH* conversion (to N* + H_2_O or OH^−^) and N*–NO coupling steps, while their TSs are inversely affected by pH. This means that the apparent activation barriers for N_2_O production at pH 14 are higher than those at pH 12.7 at low overpotentials, but lower at high overpotentials. Strikingly, it was found that N_2_ is produced via a cascade process, where the activity is not significantly determined by the catalysts used and potentials, but the local N_2_O pressure. These insights overturn our long-standing views with regard to NO electroreduction. They also suggest that highly efficient electrochemical denitrification must steer away from catalyst design to reactor optimization.

## RESULTS AND DISCUSSION

### Potential-dependent barrier calculations

**Figure 2.**
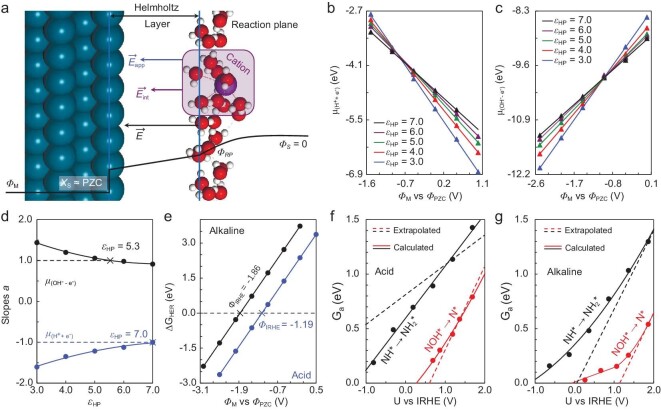
Calculations of potential-dependent energetics via the electric field controlling constant potential (EFC-CP) method. (a) Schematic model of the EFC-CP method. Chemical potentials (*μ*) of the explicit (b) proton-electron (H^+^ + e^−^) pair for the acid model and (c) (OH^−^ − e^−^) pair for the alkaline model, plotted against electrode potential vs. the potential of zero charge (*ϕ*_M_ vs. *ϕ*_PZC_). Note that *ϕ*_M_ is relative to *ϕ*_PZC_ unless specified otherwise. (d) Slopes *a* of the linear correlations between ${{\mu }_{( {{{{\mathrm{H}}}^ + } + {{{\mathrm{e}}}^ - }} )}}$ or ${{\mu }_{( {{\mathrm{O}}{{{\mathrm{H}}}^ - } - {{{\mathrm{e}}}^ - }} )}}$ and *ϕ*_M_, fitted with the dielectric constant inside the Helmholtz layer *ε*_HP_. (e) Reaction free energies of the hydrogen evolution reaction (Δ*G*_HER_) at different *ϕ*_M_, where acid and alkaline models are distinguished by blue and black lines, respectively. Potential-dependent barriers of exemplified steps in (f) acid and (g) alkaline conditions, where the calculated (EFC-CP method) and extrapolated (capacitor model) barriers are shown in solid and dotted lines, respectively.

Firstly, the electrochemical barriers involved in eNORR over Pd(111) were calculated by the EFC-CP method [37]. Electrochemical reduction reactions at acid and alkaline conditions can be expressed as A* + (H^+^ + e^−^) → AH* and A* + H_2_O → AH* + (OH^−^ − e^−^), respectively. To calculate the chemical potentials (*μ*) of (H^+^ + e^−^) and (OH^−^ − e^−^) pairs (Section 2 of the  supplementary data), monolayer water containing a hydronium (H_3_O^+δ^) and cationic group (H_2_OK^+δ^) was placed on the surface to model the acid and alkaline interfaces, respectively. The optimized structures are shown in Fig. S1. Note that there is an intrinsic field (${{\vec{E}}_{{\mathrm{int}}}}$) inside the Helmholtz layer, formed by cationic groups (or protons) and the excess charge on the electrode surface. As the proton approaches the surface, the excess charge is spontaneously transferred, resulting in a varying ${{\vec{E}}_{{\mathrm{int}}}}$ along reaction coordinates. To ensure that the apparent total field ($\vec{E}$) inside the Helmholtz layer is constant (corresponding to constant electrode potential *ϕ*_M_), as illustrated in Fig. 2a, an external field (${{\vec{E}}_{{\mathrm{app}}}}$) is applied in the EFC-CP method. ${{\vec{E}}_{{\mathrm{app}}}}$ self-adaptively varies with the structural perturbation of the water layer and electronic redistribution, and can be determined by the following equation:


(1) \begin{eqnarray*}
{{\vec{E}}_{{\mathrm{app}}}} = \vec{E} - {{\vec{E}}_{{\mathrm{int}}}}.
\end{eqnarray*}

The total field $\vec{E}$ was determined directly by required *ϕ*_M_, and the one-to-one mapping between them was given by the Bikerman-Poisson-Boltzmann (BPB) equation [38]. More details are shown in Section 3 of the supplementary  data. The intrinsic field ${{\vec{E}}_{{\mathrm{int}}}}$ is calculated by


(2) \begin{eqnarray*}
{{\vec{E}}_{{\mathrm{int}}}} = {{\vec{n}}_z}\frac{\sigma }{{{{\varepsilon }_{{\mathrm{HP}}}}}},
\end{eqnarray*}

where σ and *ε*_HP_ refer to the excess surface charge density and the dielectric constant inside the Helmholtz layer, respectively.

The *ε*_HP_ is the last undetermined parameter and depends on electrochemical interfaces and electrolytes. As the slope (*а*) of linear correlation between the chemical potentials of the (H^+^ + e^−^) pair and electrode potentials should be −1 (or 1 for the (OH^−^ − e^−^) pair) [20], this can be used as a criterion to determine *ε*_HP_. We set *ε*_HP_ with different values from 3 to 7 to calculate ${{\mu }_{( {{{{\mathrm{H}}}^ + } + {{{\mathrm{e}}}^ - }} )}}$ and ${{\mu }_{( {{\mathrm{O}}{{{\mathrm{H}}}^ - } - {{{\mathrm{e}}}^ - }} )}}$, as shown in Fig. 2b and c. The *ε*_HP_ for acid and alkaline models is determined as 7.0 and 5.3 (Fig. 2d), respectively. Note that the *ε*_HP_ will be affected by the local environment of electrochemical interfaces, such as Helmholtz layer thickness, concentration and effective diameter of interfacial ion. In other words, it is not demonstrated that the *ε*_HP_ is generally larger in the acid condition. All the reaction energies and barriers can be calculated at constant potentials vs. potential of zero charge (*ϕ*_M_ vs. *ϕ*_PZC_). In this article, *ϕ*_M_ is constantly relative to *ϕ*_PZC_ unless specified otherwise. However, experiments are usually performed at a constant RHE potential. To make a comparison with the experimental results, we defined an internal RHE (IRHE) potential (*ϕ*_IRHE_), the electrode potential at which the reaction free energy of the hydrogen evolution reaction (Δ*G*_HER_) is zero. The *ϕ*_IRHE_ at acid and alkaline conditions is −1.19 and −1.86 V vs. *ϕ*_PZC_ (Fig. 2e), respectively. By shifting *ϕ*_M_ by 1.19 (acid) and 1.86 V (alkaline), respectively, it is feasible to shift the absolute electrode potential to a relative one (*ϕ*_M_ vs. *ϕ*_IRHE_, termed U vs. IRHE), by which many systematic errors can be canceled out, too.

This method is cheaper and more practical in applications than approaches [39,40] that calculate the absolute electrode potential by *ab initio* molecular dynamics. It is also more accurate compared to the capacitor model [32,33,41]. For instance, Fig. 2f and g show the calculated (by EFC-CP method) and extrapolated (by capacitor model) barriers for two exemplified processes, NH* → NH_2_* and NOH* → N* + H_2_O or OH^−^. Note that the capacitor model assumes the adsorbed intermediates do not reorient dramatically from initial state (IS) to final state (FS). In other words, it hypothesizes that the dipole moment for intermediates does not change along with reaction coordinates. It also hypothesizes that the TS structure does not change with electrode potentials. With the two assumptions, the extrapolated barriers are linearly correlated with potentials (dotted lines) and the slope is the charge transfer coefficient (β). However, in fact, the structures of TSs and the corresponding β are dependent on potentials. The EFC-CP method can explicitly determine the real structures of TSs at varying potentials, where the contribution of adsorbate dipole to barriers is strictly considered. Thus, the correlations between calculated barriers and potentials are approximate parabolic curves (solid lines). Their slopes become smaller with increased overpotentials, reflecting the evolution of the charge transfer and the reorientation of adsorbate dipoles.

### SHE potential determination

**Figure 3.**
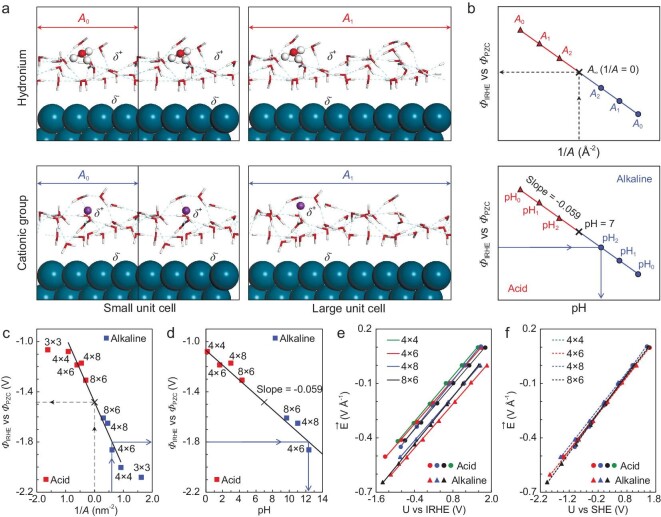
Schematic procedure of cell‐extrapolation and determination of SHE potential. (a) Explicit solvent models containing a hydronium (H_3_O^+δ^) and cationic group (H_2_OK^+δ^) in unit cells with different sizes, *A*_0_ and *A*_1_. As the unit cells increase in size, the concentrations of protons and cationic groups decrease. (b) Extrapolation of *ϕ*_IRHE_ calculated at different cell sizes *A_i_* and prediction of pH by calculating *ϕ*_IRHE_. (c) Interpolated *ϕ*_IRHE_ at infinite surface area, at pH 7. (d) Correlation between *ϕ*_IRHE_ and pH, where the scatter points represent the seven calculated models whose pH is within a range of 0 to 14. Correlations between the electric field and electrode potential versus (e) IRHE and (f) SHE.

The potential-dependent calculations were performed at IRHE potential while the further determination of SHE potential, in other words the determination of pH condition in calculation, is required for modeling pH dependence. As discussed above, the *ϕ*_IRHE_ of the cationic group model (−1.86 V) is more negative than that of the hydronium model (−1.19 V), which is qualitatively consistent with the pH effect versus potentials, but the absolute pH for the two models is not determined. Conventionally, it was estimated directly from the concentration of protons [H^+^] in the water layer. However, it has been demonstrated experimentally that the absolute [H^+^] is different between electrochemical interface and solution bulk [42,43]. Alternatively, we intend to determine the pH of computational models by the correlation between [H^+^] in solution bulk and IRHE. As shown in Fig. 3a and b, we employed a cell-extrapolation scheme to establish the quantitative correlation between computed *ϕ*_IRHE_ and solution pH. As the unit cells increase in size, the concentrations of protons and cationic groups become lower. The ion concentration at infinite cell size (*A*_∞_) corresponds approximately to the case of pH = 7. Figure 3b shows a series of hypothetical *ϕ*_IRHE_, calculated at different cell sizes *A_i_*, as a function of the reciprocal of cell size (1/*A*). It means that *ϕ*_IRHE_(pH = 7) can be determined by extrapolation to an infinite cell size. Thus, the correlation between *ϕ*_IRHE_ and pH can be determined as:


(3) \begin{eqnarray*}
{{\phi }_{{\mathrm{IRHE}}}}\!\left( {{\mathrm{pH}}} \right) = {{\phi }_{{\mathrm{IRHE}}}}\!\left( {{\mathrm{pH}} = 7} \right) - 0.059\!\left( {{\mathrm{pH}} - 7} \right).
\end{eqnarray*}

Consequently, the absolute pH value for a given cell size can be determined by its *ϕ*_IRHE_.

We built 10 explicit models with different cell sizes to obtain the interpolated *ϕ*_IRHE_ at pH 7. The calculated Δ*G*_HER_ are plotted against electrode potential *ϕ*_M_ in Fig. S2. By locating the potential of Δ*G*_HER_ = 0, the *ϕ*_IRHE_ for different models were computed and shown in Fig. 3c. There are deviations for (3 × 3) unit cells in both acid and alkaline conditions, which shows that the interfacial ion concentration for (3 × 3) unit cells is higher than dilute solution. When the unit cell is larger than (4 × 4), *ϕ*_IRHE_ approximately depends linearly on the reciprocal of the surface area (1/*A*). The interpolated *ϕ*_IRHE_(pH = 7) is −1.48 V vs. *ϕ*_PZC_. Accordingly, the correlation between *ϕ*_IRHE_ and pH values (the black solid line in Fig. 3d) can be determined. In Fig. 3d, the data points show the calculated models within a pH range of 0 to 14. Figure 3e shows the linear correlations between the electric field and electrode potential (IRHE scale). As we can see, the seven lines (Fig. 3e) collapse into a single one (Fig. 3f) as we shift the potential from IRHE to SHE scales. Thus, an approximately linear function of field against potential (SHE scale) can be determined. In addition, the [H^+^] at our interfacial models are higher than those at solution bulk (calculated by pH values). This is indeed consistent with the experimental observation [42,43] that the [H^+^] at electroreductive interfaces is higher than in bulk. Hence, this scheme is more reasonable compared to the conventional way of estimating pH directly by the [H^+^] at interfaces. The pH for a given unit cell can be easily determined by its *ϕ*_IRHE_, while it is still difficult to accurately construct the model for a given pH condition. Thus, the energetics were calculated by the (4 × 6) unit cell model (Figs S3–S10), where pH was determined as 1.6 and 12.4 for acid and alkaline, respectively, and finally extrapolated to pH 0.5, 12.7 and 14 (Section 4 in the supplementary  data).

### Comparison between acid (pH 0.5) and alkaline (pH 12.7)

For both N_2_O and N_2_ production, the current densities in alkaline solutions (pH 12.7) are generally higher than those in acid conditions (pH 0.5), as shown in Fig. 1. To analyze this, we considered the thermodynamically optimal path (NO → NOH* → N* + NO → N_2_O, Table S1) on Pd [6] and built a pH-dependent microkinetic model (the details are shown in Section 5 of the supplementary  data). In addition, at positive potentials, ammonia is a by-product with low Faradaic efficiency (Fig. S11). Theoretically, NH_4_^+^ production indeed has higher barriers in limiting steps than N_2_O on Pd (Fig. S12). Hence, it will not be discussed in the analysis of pH dependence.

**Figure 4.**
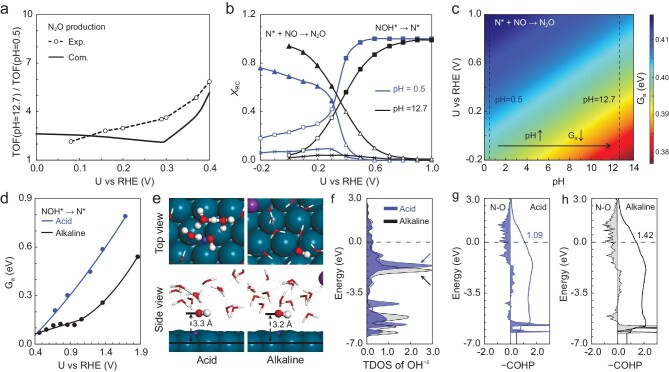
Comparison of N_2_O production in acid (pH 0.5) and alkaline (pH 12.7) conditions. (a) Quantitative comparison between simulations and experiments. (b) Degree of rate control (DRC) for different elementary steps (*X*_RC_), where the rate-determining steps (RDSs) are represented by solid points. (c) Evolution of the N*–NO coupling barrier along coordinates of potential and pH. (d) Barrier comparison for conversion of NOH* to N* + H_2_O or OH^−^. (e) Structures of transition states (TSs) for NOH* → N* step calculated by the explicit solvent model of hydronium and cationic groups. (f) Total density of states (TDOS) for OH^−δ^ in TSs. Analyzing crystal orbital Hamilton population (COHP) for the N–O bond of TSs calculated by (g) acid and (h) alkaline models. The filled shadow and solid lines are −COHP and −ICOHP (integrated crystal orbital Hamilton population), respectively.

As shown in Fig. S13, in the wide potential region from 0.8 to −0.2 V vs. RHE, the intrinsic activity for N_2_O production at pH 12.7 is higher than that at pH 0.5. It is consistent with the experimental trends (Fig. S14b). For further quantitative comparison between simulations and experiments, we calculated the ratio of TOF at pH 12.7 to that at pH 0.5. Note that the experiments were performed with the mode of constant current density and there were small deviations in potentials for the two pHs. As the comparison between experimental and theoretical results should be performed at constant potentials, the fitted current densities were used to compute the ratio of experimental reaction rate. As shown in Fig. 4a, the computational production of N_2_O (solid line) at pH 12.7 increases by 2–6 times compared to that at pH 0.5, which is in quite acceptable agreement with experiments (dotted line).

To understand the enhanced activity of N_2_O production in alkaline solutions, the degree of rate control (DRC) [44,45] for elementary steps (*X*_RC_), which reflects the importance (weight) of a given step on total reaction rate, was analyzed and the results are shown in Fig. 4b. The process with the largest *X*_RC_ is defined as the rate-determining step (RDS). At high overpotentials, N*–NO coupling (N* + NO → N_2_O) limits the reaction rate. As N* is not sensitive to the electric field (Fig. S10), while the TS can be stabilized by a negative field (Fig. S10), it effectively leads to a decrease in barriers with increased pH (Fig. 4c) and thus the promotion of N_2_O production activity. At low overpotentials, the RDS is NOH* conversion to N* + H_2_O or OH^−^. The enhanced activity can be attributed to assisted (geometrical) effects on NOH* conversion by the cationic group, as reflected by the lower barriers in alkaline conditions (Fig. 4d). In alkaline conditions, the NOH* → N* + (OH^−^ − e^−^) step goes through the direct breaking of the N–O bond, while an additional proton transfer is required in acid solution. As a result, the formed OH^−δ^ at TS (alkaline) is closer to the electrode surface (Fig. 4e). Figure 4f shows the total density of states (TDOS) for OH^−δ^ at TS, where the deeper and more stable electronic states indicate its higher stability with the cationic group. According to the analysis of crystal orbital Hamilton population (COHP) for the N–O bonding interaction (Fig. 4g and h), the larger −ICOHP at Fermi level (1.42 versus 1.09) suggests that the stabilization can also be reflected by the stronger electronic interaction between OH^−δ^ and N*. In short, the positive dipole moment of the TS for N*–NO coupling and the assisted (geometrical) effects of cationic group on NOH* conversion give rise to the enhancement of eNORR in alkaline conditions, compared to acid.

### pH dependence of N_2_O selectivity in alkaline

**Figure 5.**
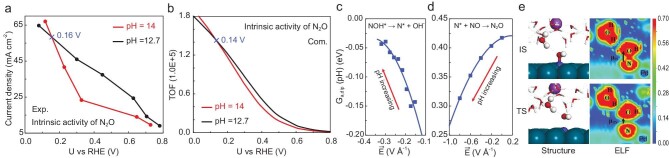
Insights into the reverse of N_2_O activity between pH 14 and 12.7. (a) Intrinsic current density for N_2_O production at pH 14 and 12.7. (b) Computational reaction rate for N_2_O at pH 14 and 12.7. Evolution of pH-dependent contributions from the adsorbate dipole to the barrier, namely *G*_a, dip_(pH), for (c) NOH* → N* + (OH^−^ − e^−^) and (d) N* + NO → N_2_O steps. (e) Structures and electronic localization functions (ELFs) for IS and TS.

There is an interesting reversal from pH 12.7 to pH 14 for the intrinsic activity of N_2_O production, where the crossover potential is ∼0.16 V vs. RHE (Fig. 5a). Note that there is a small difference between Figs 1b and 5a. Please see more details with regard to the recalculations of partial current densities in Fig. S14. We performed a microkinetic simulation with calculated barriers and successfully reproduced this reversal, as shown in Fig. 5b. Two critical factors are identified for the activity reversal. The first factor is that the RDS for N_2_O production switches from NOH* → N* + (OH^−^ − e^−^) to N*–NO coupling with increased overpotentials. However, the electric field effect is exactly inverse for the two steps, as shown in Fig. 5c and d. This is the second influencing factor. The two synergistic factors mean that the apparent activation barrier for N_2_O production at pH 14 is higher at low overpotentials, but lower at high overpotentials, compared with pH 12.7 (Fig. S15). For the NOH* → N* + (OH^−^ − e^−^) step, the pH-dependent contributions of adsorbate dipole to barrier, namely *G*_a, dip_(pH), become higher with increasing pH (Fig. 5c), where the chemical origin is the dipole reorientation of NOH* species. As shown in Fig. 5e, the positive dipole moment of NOH* species diminishes and even disappears due to the N–O bond breaking from IS to TS.

### pH dependence of N_2_ selectivity in alkaline

**Figure 6.**
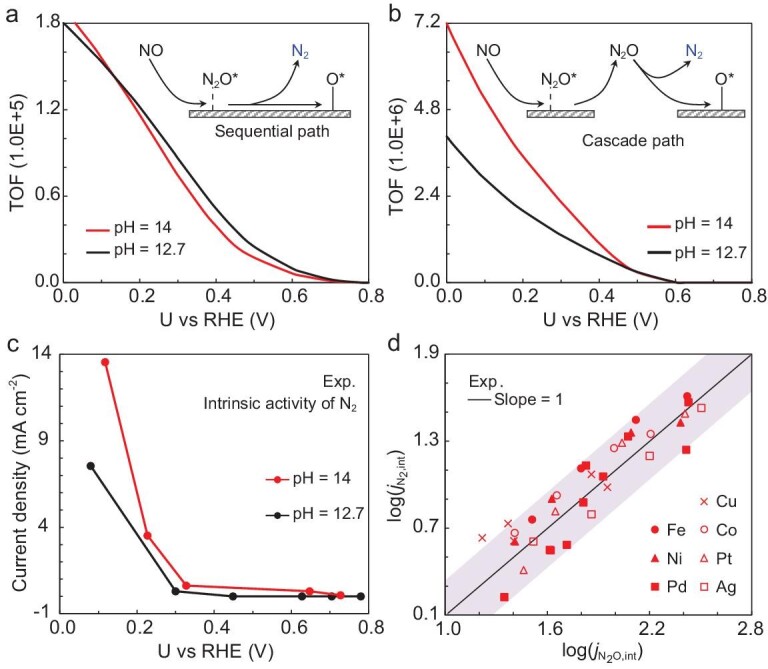
Mechanistic investigation for N_2_ production. Activity trends for N_2_ production simulated via (a) sequential and (b) cascade mechanisms. (c) Intrinsic current density of N_2_ production. (d) Relationship between experimental $\log ({{j}_{{{{\mathrm{N}}}_2},{\mathop{\mathrm{int}}} }})$ and $\log ({{j}_{{{{\mathrm{N}}}_2}{\mathrm{O}},{\mathop{\mathrm{int}}} }})$ [5], with small deviations of ±0.25.

Discussions about the pH dependence of N_2_O production confirm that the presented scheme of modeling pH dependence is reliable. We now turn to investigating the mechanism for N_2_ production and its pH dependence. There are two possible major reaction paths for N_2_ production (Table S1). First, the N_2_ can be produced from intermediates, such as N_2_O* [6], which is defined as the sequential path (NO → NOH* → N* + NO → N_2_O* → N_2_ + O* → OH* → H_2_O). Second, the N_2_ may be produced from desorbed N_2_O gas. In other words, the N_2_O* was first desorbed and followed by direct dissociation to N_2_ + O* via secondary conversion. This is also a reasonable assumption as the N_2_O adsorption is very weak. For the sequential mechanism, N_2_ and N_2_O production share many key steps (NO → NOH* → N* + NO → N_2_O*). The behavior of N_2_ production (Fig. 6a) at pH 14 and 12.7 should be identical to that of N_2_O production (Fig. 5b). However, as we have shown in Fig. 1b and c, the pH dependence of N_2_ and N_2_O production is completely different. Thus, we speculate that N_2_ is likely to be produced indirectly from main-product N_2_O, rather than directly from original reactant NO.

N_2_ production by a cascade mechanism has relatively small barriers (<0.35 eV) (Fig. S16b) and extremely low N_2_O pressure (<0.01 atm) due to the low NO conversion. This may lead to the N_2_ production rate being more sensitive to the partial pressure of N_2_O. Therefore, the N_2_O pressure effect on the activity of N_2_ production was firstly examined, as the pH increased from 12.7 to 14. At pH 12.7, we estimated N_2_O pressure by the partial current densities (the intrinsic values in Fig. S10). At pH 14, the partial pressure of N_2_O was approximately set to *m* times that at pH 12.7. As shown in Fig. S17a, at 0.2 V vs. RHE, the activity of N_2_ production at pH 14 is higher than that at pH 12.7 (consistent with experiments). More importantly, the difference in N_2_ production rate between pH 14 and pH 12.7 becomes larger, with increased *m* (N_2_O pressure at pH 14). This indicates the high dependence of N_2_O pressure on the activity of N_2_ production. Furthermore, at varying potentials, Fig. 6b and Fig. S17b again show consistent trends with experimental results (Fig. 6c). Note that higher N_2_O pressures at pH 14 is a reasonable assumption. As pH increases, the electric field becomes more negative, which can stabilize N_2_O physical adsorption. In other words, the lifetime of N_2_O at the electrochemical interface will increase. Therefore, the higher activity of N_2_ production at pH 14 is primarily caused by the higher local pressure/concentration of N_2_O.

Experimentally, the slope of the correlation between $\log ({{j}_{{{{\mathrm{N}}}_2},{\mathop{\mathrm{int}}} }})$ and $\log ({{j}_{{{{\mathrm{N}}}_2}{\mathrm{O}},{\mathop{\mathrm{int}}} }})$ approaches 1, in spite of the varying potentials (from −0.2 to 0.2 V vs. RHE), NO pressures (25%, 50% and 100%) and catalysts used (Pd, Fe, Ni, Cu, Co, Pt, Ag), as shown in Fig. 6d. This means, over all catalysts, the apparent activation barriers for N_2_ production should be identical to those for N_2_O, if the sequential path dominates N_2_ production. In other words, the two products should share the same RDS and thus show similar pH dependence. However, over Pd, the experimental results show obviously different pH dependence between them. By assuming N_2_O* is firstly desorbed and followed by direct dissociation to N_2_, such a cascade mechanism can better explain experiments. This is a reasonable assumption as N_2_O* adsorption on Pd is very weak (+0.18 eV). Likewise, our previous work [6] has proved the constantly weak adsorption of N_2_O* over transition metals, with positive adsorption free energies (0 < *G*_ad_N_2_O* < +0.3, in eV, Table S2). Therefore, we propose that the dominant cascade path on Pd can be generalized to other metals. In addition, we do not exclude the contribution of the sequential mechanism. We think the contribution of the sequential path will be one of the key factors for the deviations in Fig. 6d. Besides, the deviations are also associated with variations in the local pressure/concentration of N_2_O, caused by interfacial fluctuations.

In a word, the statistical correlation again indicates that N_2_ production heavily depends on N_2_O yield, which further confirms the rationality of the cascade mechanism. Indeed, in previous work, there are some experimental conditions, involving for instance, a larger surface area of electrodes and lower NO flow rate, in which the Faradaic efficiency of N_2_ production can be significantly enhanced [46–48]. This can be attributed to facilitated secondary conversion of as-produced N_2_O. Therefore, the findings above suggest that it is a better strategy to make efforts with reactor optimization rather than catalyst design.

## CONCLUSION

There is a lot of debate about the origin of the pH dependence of electrocatalysis in general. Herein, we have presented a cell-extrapolated scheme to determine SHE potential based on the EFC-CP method, which allows us to accurately model the pH dependence of electrochemical barriers for the CPET process, with an electric field. The simulated pH-dependent activities are in good agreement with experiments. We found that the activity for eNORR depends on two RDSs. The first is the formation of the N*–NO bond. Its transition state can be stabilized by negative field (high pH) due to the positive dipole moment. The second RDS is NOH* conversion to N* + H_2_O or OH^−^, which can be facilitated by the assisted (geometrical) effect of the cationic group. The two natures collectively lead to the enhancement of N_2_O production in alkaline conditions. However, N_2_O production is not constantly promoted with increasing pH, because the positive dipole moment of N–O bonds diminishes and even disappears from IS to TS. This means the dipole of NOH* species reorients dramatically, giving rise to a higher barrier and thus lower activity at pH 14 than pH 12.7. Finally, for N_2_ production, the cascade mechanism shows better agreement with experiments than the sequential mechanism. This indicates that N_2_ was primarily produced from as-produced N_2_O, rather than directly from original reactant NO. This insight suggests that electrochemical denitrification should focus on reactor design, for example with regard to a cascade reactor, rather than catalyst design.

## Supplementary Material

nwae147_Supplemental_File
