# Supplementary material for: Towards rational design in electrochemical denitrification by analyzing pH dependence
Source: Natl Sci Rev. 2024 Apr 16;11(6):nwae147. doi: 10.1093/nsr/nwae147 (PMC11092274; doi:10.1093/nsr/nwae147)
Supplement: nwae147_Supplemental_File [file nwae147_supplemental_file.pdf]

Supporting information for

Towards rational design in electrochemical denitrification by analyzing pH-dependence

Huan Li[1,2], Dong Luan[1], Jun Long[1], Xiaoyan Fu[1], Jianping Xiao*[1,2]

[1] State Key Laboratory of Catalysis, Dalian Institute of Chemical Physics, Dalian National Laboratory for Clean Energy, Chinese Academy of Sciences, Dalian 116023, P. R. China.

[2] University of Chinese Academy of Sciences, Beijing 100049, P. R. China.

* Corresponding author: xiao@dicp.ac.cn

Section 1. Computational details

The Vienna *ab initio* Simulation Package (VASP)[1-3] was used to conduct density functional theory (DFT) calculations, where the revised Perdew-Burke-Ernzerhof (rPBE)[4] functional under generalized gradient approximation (GGA)[5] was used. We applied the projected augmented wave (PAW)[6,7] potentials to describe core electrons, and set the kinetic energy cutoff to 400 eV to describe valance electrons. The convergence criterion in structural optimizations consisted of the residual force and electronic energy differences that were set to −0.05 eV/Å and 10^{-5} eV, respectively. Then, the obtained electronic energies were corrected by zero-point energy and entropic (S) contribution[8], at the experimental temperature (T = 298K). In terms of entropic contribution, we considered vibrational entropy for adsorbed intermediates while translational, rotational, and vibrational motions for gases, respectively. Referenced to gaseous N_2, H_2 and NO, the adsorption free energies for adsorbed intermediates were evaluated using four-layer Pd(111) surfaces, with the bottom two layers fixed. The scheme of Methfessel-Paxton[9] (N = 1) with width of 0.2 eV was used for smearing.

The most of potential-dependent energetics were calculated using (4×6) supercell, where a 2×2×1 Monkhorst–Pack *k*-point grid was used. To determine the standard hydrogen electrode (SHE), additional models with different cell size, such as (3×3), (4×4), (4×8) and (8×6), were constructed, where 4×4×1, 3×3×1, 2×2×1, 2×2×1 Monkhorst–Pack *k*-point grids were used, respectively. Besides, we placed a monolayer water containing a hydronium ($H_3O^{+\delta}$) or cationic group ($H_2OK^{+\delta}$) on those surfaces to explicitly model the acid and alkaline interfaces, respectively. For a given coupled proton-electron transfer (CPET) process, locating the real structures of transition states at different electrode potential was performed by the electric field controlling constant potential (EFC-CP) method. More details for this approach can be referred to our previous work[10]. To predict the response of thermochemical steps to field (potential and pH), their transition states were searched with applied electric field, which was applied using a saw-tooth potential corresponding to fields between −0.8 and 0.8VÅ^{-1}.

Section 2. Chemical potentials (μ) of (H^{+} + e^{-}) and (OH^{-} − e^{-}) pairs

Electrochemical reduction reactions at acid and alkaline (neutral) conditions can be expressed as A* + (H^{+} + e^{-}) → AH* and A* + H_2O → AH* + (OH^{-} − e^{-}), respectively. The key for potential-dependent calculations is to describe the chemical potentials of (H^{+} + e^{-}) and (OH^{-} − e^{-}) pairs, which were calculated by the following equations:

$$\mu_{(\mathrm{H^{+}+e^{-}})} = E(\mathrm{H_3O^{+}}) - E(\mathrm{H_2Os}) - \Delta E^{\mathrm{corr,ac}} \quad \text{(S1)}$$

$$\mu_{(\mathrm{OH^{-}-e^{-}})} = E(\mathrm{HOK}) + E(\mathrm{H_2O}) - E(\mathrm{H_2OK^{+}}) + \Delta E^{\mathrm{corr,alk}} \quad \text{(S2)}$$

$E(H_3O^{+})$, $E(H_2Os)$, E(HOK), and $E(H_2OK^{+})$ are the total energies of the four configurations in **Fig. S1**. $\Delta E^{\mathrm{corr,ac}}$ and $\Delta E^{\mathrm{corr,alk}}$ refer to the energy corrections for proton (H^{+}) transfer from solution bulk to the acid interface ($H^{+\delta}$) and $OH^{-\delta}$ transfer from alkaline interface to solution bulk (OH^{-}), respectively. The general computational formula of this energy correction is

$$\Delta E^{\mathrm{corr}} = (1-\delta)(\phi_{\mathrm{M}} - \phi_{\mathrm{PZC}}) + \delta\phi_{\mathrm{RP}} \quad \text{(S3)}$$

The potential of zero charge (PZC) is represented by ϕ_{PZC}, and ϕ_{RP} refers to the potential at reaction plane (**Fig. 2a**). For convenience, we set ϕ_{PZC} as the reference in all DFT calculations. It means ϕ_{M} is referenced to ϕ_{PZC} in this work unless specified otherwise.

Section 3. Determining the one-to-one correspondence between $\vec{E}$ and ϕ_M

In this work, we employed the Stern electric double layer model to determine the one-to-one correspondence between electric field $\vec{E}$ and electrode potential ϕ_M, which is given by Bikerman-Poisson-Boltzmann (BPB) equation[11]. As described in our previous work[10], there are six important parameters in the BPB equation: temperature T, the concentration of anions (cations) in the bulk solution c_b, the finite volume of ion d_t^3, the dielectric constant inside the Helmholtz layer ε_{HP}, the thickness between electrode surface and Helmholtz plane δ_{HP}, and the position of the solution bulk X_∞. As T, c_b, and X_∞ have small effect on $\vec{E}$, they were set to fixed values: $T = 298.15$ K, $c_b = 0.01$ mol/L, and $X_\infty = 80\ \lambda_D$, where λ_D refers to Debye length. Besides, we set d_t to a moderate value of 10.0 Å. δ_{HP} was defined as the distance between the electrode surface and the water layer, and can be directly determined by the explicit solvent model. The last one undetermined parameter, ε_{HP}, is related to the electrochemical interfacial environment. Herein, we get ε_{HP} by the fitting method as introduced in main text.

Section 4. Energetic extrapolation to a given pH value

With the procedure of cell-extrapolation, the pH for a given unit cell can be determined by its ϕ_{IRHE}, while it is still difficult to accurately construct the model for a given pH. Thus, the most of potential-dependent energetics were calculated using (4×6) unit cell model (**Fig. S3** to **S10**), where pH is determined as 1.6 and 12.4 for acid and alkaline conditions, respectively. We extrapolated those energies to pH = 0.5, 12.7 and 14 with an electric field effect, as it is the important contribution to pH effect[12-15]. For an electroreduction step, A* + (H^+ + e^-) → TS → AH*, from state 1 to 2, where state 1 is IS and state 2 corresponds to TS or FS, the energy change is

$$\Delta E = E_{\text{ads},2} - E_{\text{ads},1} - \mu_{(\text{H}^+ + \text{e}^-)} \tag{S4}$$

At IRHE potentials, the chemical potential of proton-electron pair is

$$\mu_{(\text{H}^+ + \text{e}^-)} = \frac{1}{2}E_{\text{H}_2} + (q_2 - q_1)U_{\text{IRHE}} \tag{S5}$$

When adsorbates dipoles reorientate dramatically from state 1 to 2, Eq. S4 can be expressed as

$$\Delta E = \left(E_{\text{ads},2}^{\text{PZC}} - E_{\text{ads},1}^{\text{PZC}} - \frac{1}{2}E_{\text{H}_2}\right) + \left\{(\mu_2 - \mu_1)\vec{E} - \frac{\alpha_2 - \alpha_1}{2}\vec{E}^2\right\} - (q_2 - q_1)U_{\text{IRHE}} \tag{S6}$$

Here, q_i, μ_i and α_i (i = 1, 2) represent the charge of proton, the intrinsic dipole moment and polarizability of adsorbates, respectively. Note that we assume Eq. S6 is conditionally effective when state 2 corresponds to TS without obviously structure change of adsorbates. In other word, Eq. S6 can be used to calculate the pH dependence on electrochemical barriers in relatively narrow potential region.

When viewed on IRHE scale, the capacitor energy, namely ΔE_{cap}, is pH independent (Eq. S7), while the other contributions of adsorbate dipole to energy change is pH-dependent. We defined this pH-dependent contributions as ΔE_{dip}(pH) (Eq. S8). Hence, Eq. S6 has new expression as Eq. S9.

$$\Delta E_{\text{cap}} = -(q_2 - q_1)U_{\text{IRHE}} \tag{S7}$$

$$\Delta E_{\text{dip}}(\text{pH}) = \left(E_{\text{ads},2}^{\text{PZC}} - E_{\text{ads},1}^{\text{PZC}} - \frac{1}{2}E_{\text{H}_2}\right) + \left\{(\mu_2 - \mu_1)\vec{E}(\text{pH}) - \frac{\alpha_2 - \alpha_1}{2}\left[\vec{E}(\text{pH})\right]^2\right\} \tag{S8}$$

$$\Delta E(\text{pH}) = \Delta E_{\text{dip}}(\text{pH}) + \Delta E_{\text{cap}} \tag{S9}$$

The electric field $\vec{E}$ can be uniquely determined using U_{SHE} (or by U_{IRHE} and pH).

Fig. 3f shows the linear function of $\vec{E}$ against U_{SHE} ($\vec{E} = 0.21U_{\text{SHE}} - 0.22$). Thus, Eq. S8 has new expression as Eq. S10.

$$\Delta E_{\text{dip}}(\text{pH}) = \left\{(\mu_2 - \mu_1)[0.21U_{\text{SHE}}(\text{pH}) - 0.22] - \frac{\alpha_2 - \alpha_1}{2}[0.21U_{\text{SHE}}(\text{pH}) - 0.22]^2\right\} + \left(E_{\text{ads},2}^{\text{PZC}} - E_{\text{ads},1}^{\text{PZC}} - \frac{1}{2}E_{\text{H}_2}\right) \quad \text{(S10)}$$

A second-order polynomial was fitted between calculated ΔE_{dip}(pH) and U_{SHE}. Then, the reaction free energies and barriers calculated by (4×6) supercell (pH = 1.6 and 12.4) can be extrapolated to a given pH, such as 0.5, 12.7 and 14.

Taking O* + (H^+ + e^-) → OH* as an example, we now introduce our procedure for extrapolating energetics to a given pH. It goes in three steps:

1. We firstly calculated the reaction energies ΔG(pH=1.6) and barriers G_a(pH=1.6) using (4×6) supercell, at several IRHE potentials, such as 1.19, 0.69, 0.19, −0.31, −0.81 and −1.31 V. According to Eq. S7 and S9, ΔG_{cap}, ΔG_{dip}(pH=1.6) and $G_{\text{a,cap}}$, $G_{\text{a,dip}}$(pH=1.6) can be computed by

$$\Delta G_{\text{cap}} = U_{\text{IRHE}} \quad \text{(S11)}$$

$$\Delta G_{\text{dip}}(\text{pH=1.6}) = \Delta G(\text{pH=1.6}) - \Delta G_{\text{cap}} \quad \text{(S12)}$$

$$G_{\text{a,cap}} = \beta U_{\text{IRHE}} \quad \text{(S13)}$$

$$G_{\text{a,dip}}(\text{pH=1.6}) = G_{\text{a}}(\text{pH=1.6}) - G_{\text{a,cap}} \quad \text{(S14)}$$

The results are listed in the following table:

U_{IRHE} (V)	1.19	0.69	0.19	−0.31	−0.81	−1.31
U_{SHE} (V)	1.09	0.59	0.09	−0.41	−0.91	−1.41
ΔG(pH=1.6)	0.46	0.02	−0.41	−0.86	−1.30	−1.76
ΔG_{cap}	1.19	0.69	0.19	−0.31	−0.81	−1.31
ΔG_{dip}(pH=1.6)	−0.73	−0.67	−0.60	−0.55	−0.49	−0.45
G_{a}(pH=1.6)	0.62	0.51	0.42	0.32	0.20	0.09
β	0.26	0.05	0.04	0.04	0.05	0.04
$G_{\text{a,cap}}$	0.31	0.03	0.01	−0.01	−0.04	−0.05
$G_{\text{a,dip}}$(pH=1.6)	0.31	0.48	0.41	0.33	0.24	0.14

2. As shown in **Fig. S18**, the pH-dependent contributions to energy, namely, $\Delta G_{dip}(pH=1.6)$ and $G_{a,dip}(pH=1.6)$, were fitted against U_{SHE}, with a second-order polynomial. The obtained two functions are

$$\Delta G_{dip}(pH=1.6) = -0.01U_{SHE}^2 - 0.12U_{SHE} - 0.59 \tag{S15}$$

$$G_{a,dip}(pH=1.6) = -0.02U_{SHE}^2 + 0.16U_{SHE} + 0.40 \tag{S16}$$

Note that as potential (IRHE) decrease from 1.19 to 0.69 V, the TS changes from a middle structure to IS-like structure, and it almost keeps intact when potential is lower than 0.69 V, as shown in **Fig. S5**. Thus, Eq. S16 is effective in the potential region from 0.69 to −1.31 V vs IRHE.

3. At experimental RHE potential region (for example, from 0.6 to 0.0 V), Eq. S15 and S16 have new expressions as pH change from 1.6 to another value (for example 0.5):

$$\Delta G_{dip}(pH=0.5) = -0.01(U_{RHE} - 0.0591\times0.5)^2 - 0.12(U_{RHE} - 0.0591\times0.5) - 0.59 \tag{S17}$$

$$G_{a,dip}(pH=0.5) = -0.02(U_{RHE} - 0.0591\times0.5)^2 + 0.16(U_{RHE} - 0.0591\times0.5) + 0.40 \tag{S18}$$

Then, the extrapolated reaction energies and barriers can be computed by

$$\Delta G(pH=0.5) = \Delta G_{cap} + \Delta G_{dip}(pH=0.5) \tag{S19}$$

$$G_a(pH=0.5) = G_{a,cap} + G_{a.dip}(pH=0.5) \tag{S20}$$

Section 5. Microkinetic modeling

The pH-dependent microkinetic modeling towards N_2O and N_2 on Pd was performed using the CATKINAS package[16], developed by JianFu Chen and HaiFeng Wang. The rates for elementary reactions were calculated by

$$\text{rate(pH)} = k_f(\text{pH}) \prod \theta_{\text{reac}}(\text{pH}) - k_b(\text{pH}) \prod \theta_{\text{prod}}(\text{pH}) \tag{S21}$$

where the coverages of reactants and products are represented by θ_{reac} and θ_{prod}, respectively. The degree of difficulty for forward and backward reactions are reflected by reaction constants, namely k_f and k_b, respectively, which was calculated using Arrhenius equation:

$$k(\text{pH}) = Ae^{-\frac{G_a(\text{pH})}{k_B T}} \tag{S22}$$

where A (S^{-1}), G_a, k_B and T refer to reaction prefactor, activation free energy, Boltzmann constant and reaction temperature, respectively.

We performed the pH-dependent microkinetic modeling at 298 K and potential region from 0.8 to −0.2V vs RHE. We used a constant partial pressure of 0.1 atm for reactant NO at all studied pH and potentials as its solubility is very low in aqueous solution. As another reactant, the hydrogen donor prefers to be proton in acid conditions and water in alkaline (neutral) solutions. At pH = 0.5, the concentration of proton was set to 0.32 mol/L. In alkaline conditions (pH = 12.7 and 14), the concentration of reactant water at electrochemical interface was set as the cation concentration in bulk solution (1 mol/L). Note that there is a OH^- produced during hydrogenation and the concentrations of OH^- were set to 0.05 and 1 mol/L in pH 12.7 and 14, respectively. The partial pressures of products N_2O and N_2 at interface are related to reaction conditions (such as potential and pH), so that they were estimated by the experimentally measured partial current densities (the intrinsic values in **Fig. S10**) at different potentials and pH. Additionally, in order to studying the mechanism of N_2 production, we proposed a new cascade path apart from the traditional sequential path (**Table S1**), where we approximatively set the N_2O pressure at pH = 14 to m times that at pH = 12.7. More details see **Fig. S17**.

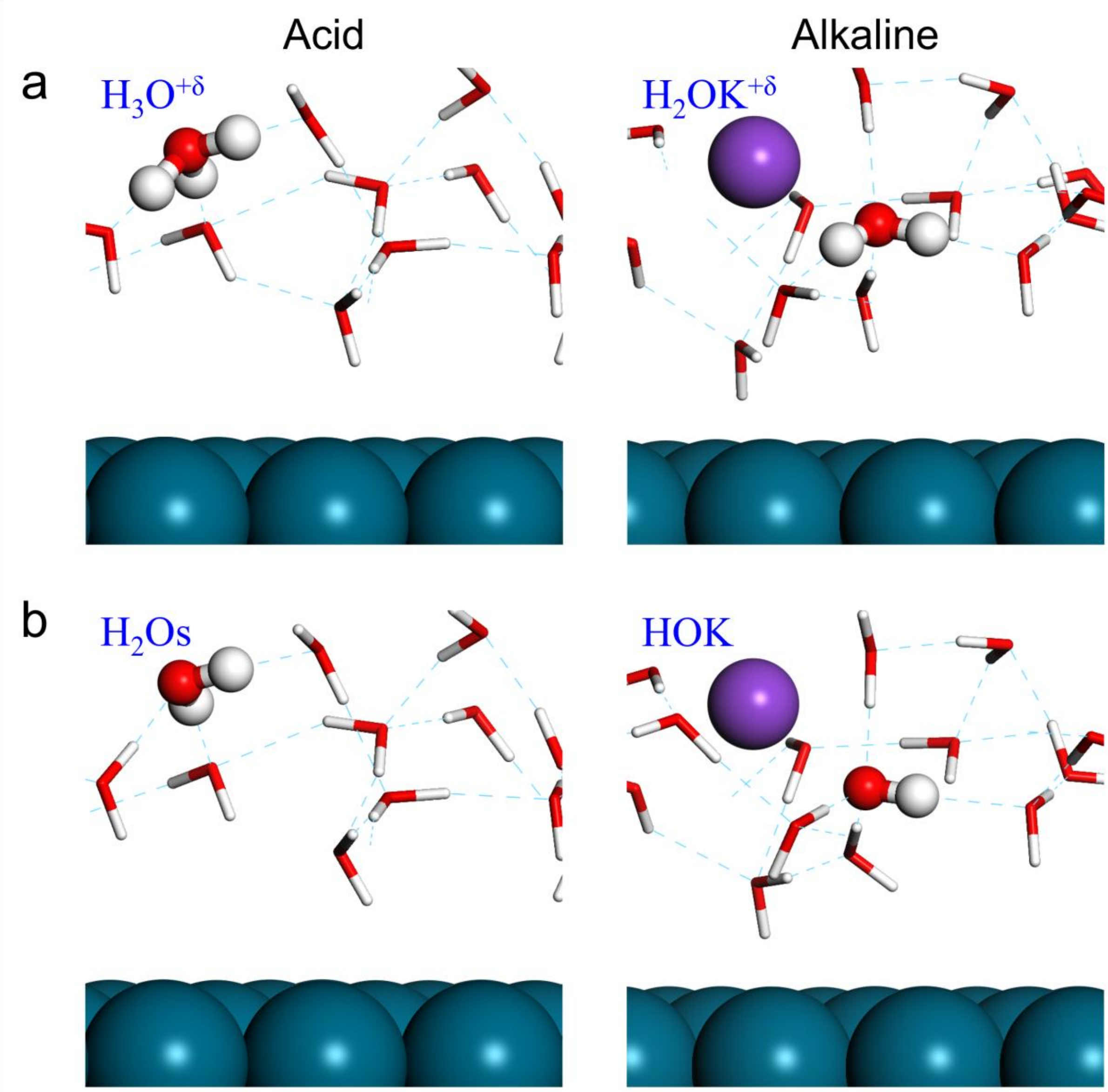

Fig. S1 Optimized structures of explicit solvent models, (**a**) $H_3O^{+\delta}$ and $H_2OK^{+\delta}$, (**b**) H_2Os and HOK, where H_2Os and HOK refer to the final states after protonation for H_3O^+ and H_2OK^+.

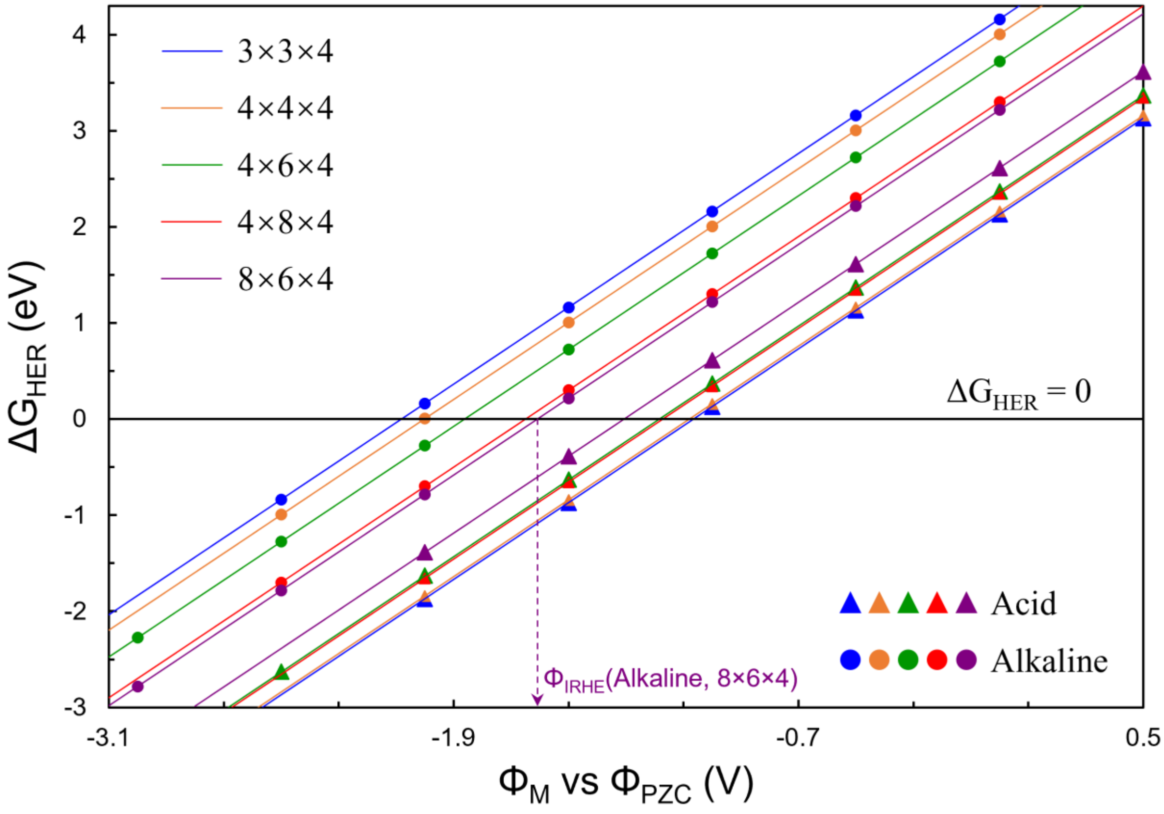

Fig. S2 Reaction free energies of hydrogen evolution reaction (ΔG_{HER}) at different electrode potentials, calculated by ten explicit solvent models. The purple dotted arrow represents an example, namely (8×6×4) supercell with alkaline solvation, for how to determine the ϕ_{IRHE} for a given unit cell.

Note:

The ϕ_{IRHE} for models with different size were computed by locating the potential of $\Delta G_{HER} = 0$. Extrapolating the cell size to infinite case, ϕ_{IRHE}(pH=7) can be determined. Then, according to Nernst equation, the absolute pH for a given cell size can be calculated via its ϕ_{IRHE}.

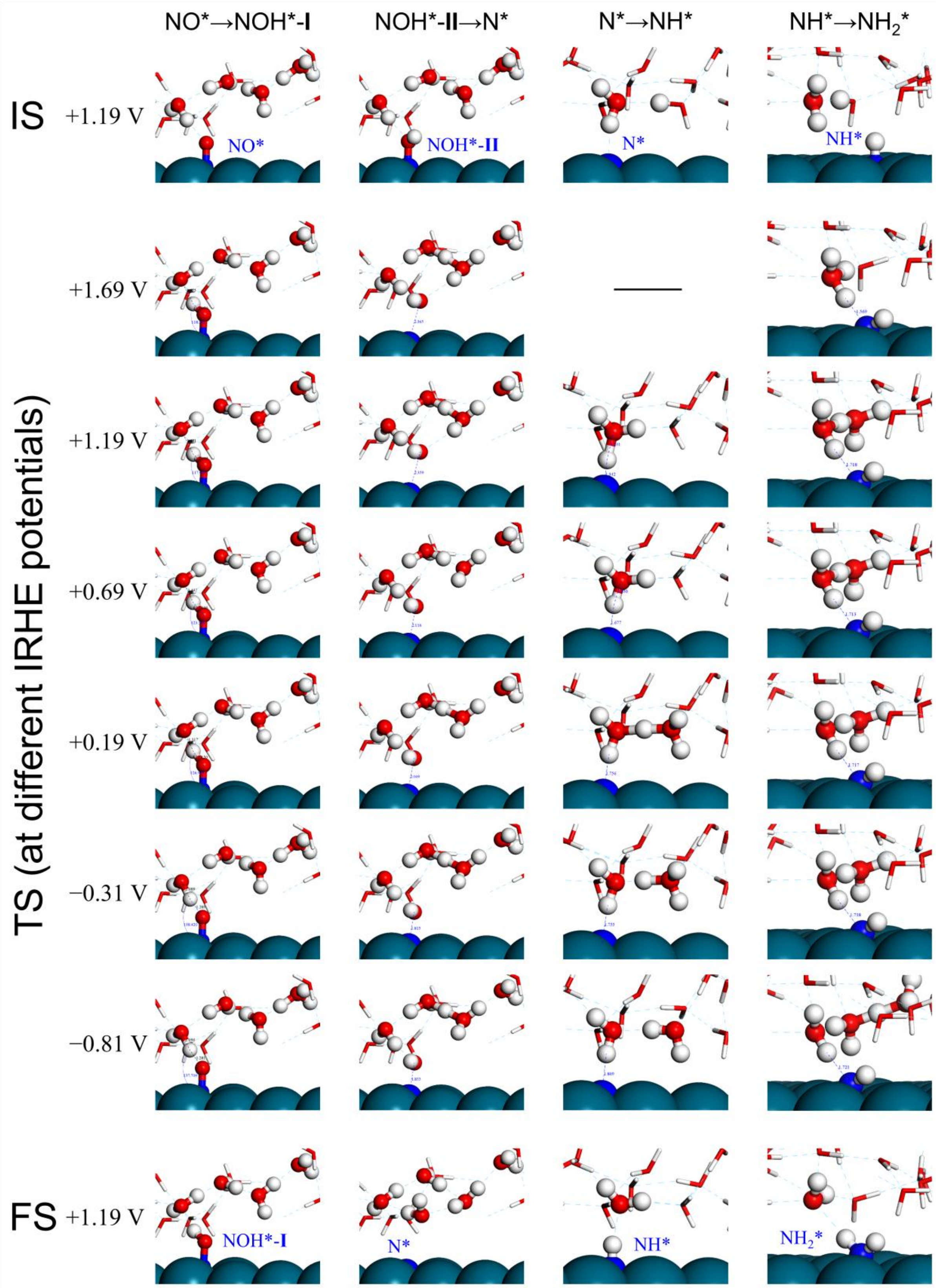

Fig. S3 Evolution of transition states for electrochemical steps: NO* + (H^+ + e^-) → NOH*-I, NOH*-II + (H^+ + e^-) → N* + H_2O, N* + (H^+ + e^-) → NH* and NH* + (H^+ + e^-) → NH_2*, with increased overpotentials, in acid conditions.

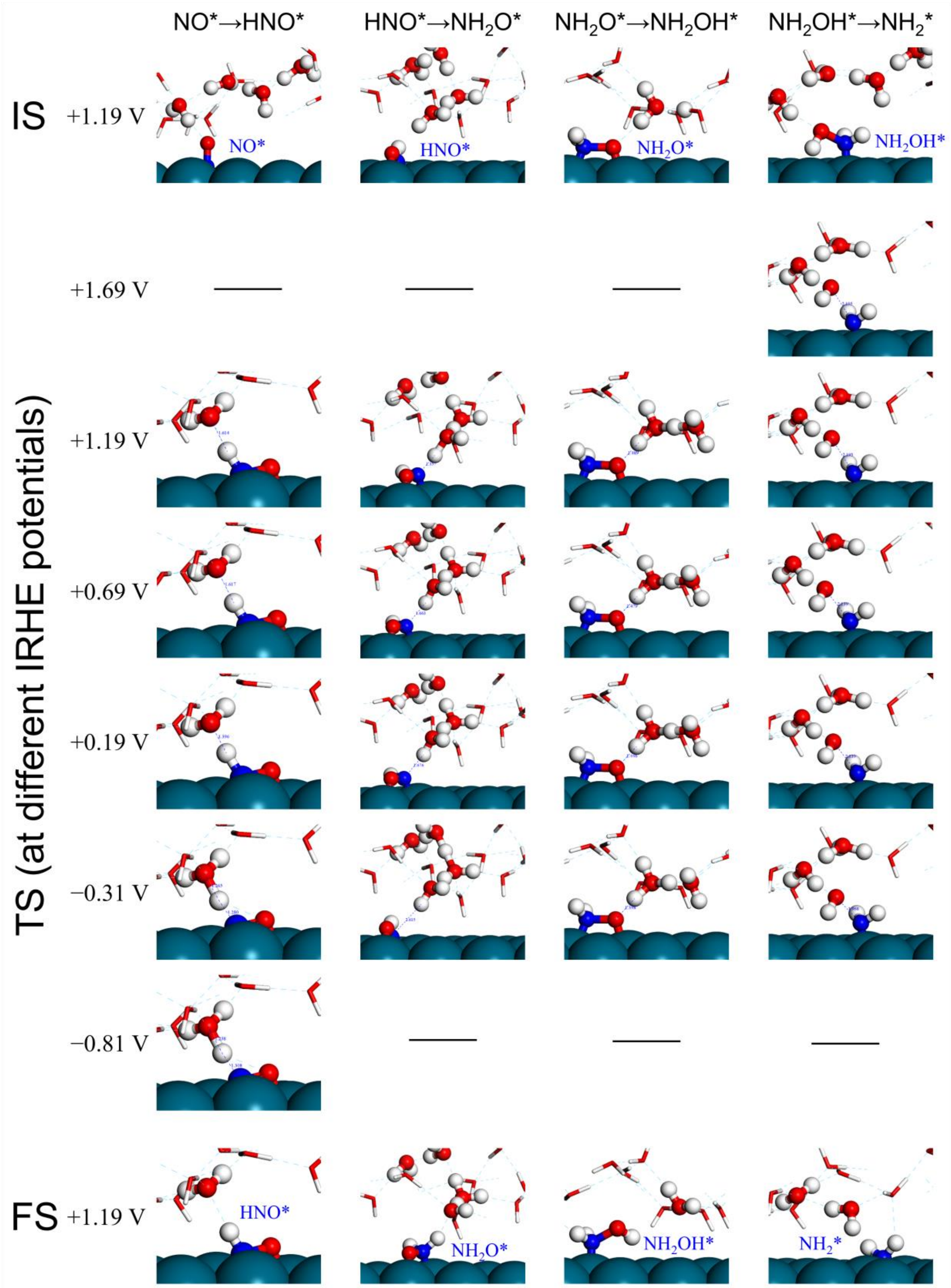

Fig. S4 Evolution of transition states for electrochemical steps: $NO^* + (H^+ + e^-) \rightarrow HNO^*$, $HNO^* + (H^+ + e^-) \rightarrow NH_2O^* + H_2O$, $NH_2O^* + (H^+ + e^-) \rightarrow NH_2OH^*$ and $NH_2OH^* + (H^+ + e^-) \rightarrow NH_2^* + H_2O$, with increased overpotentials, in acid conditions.

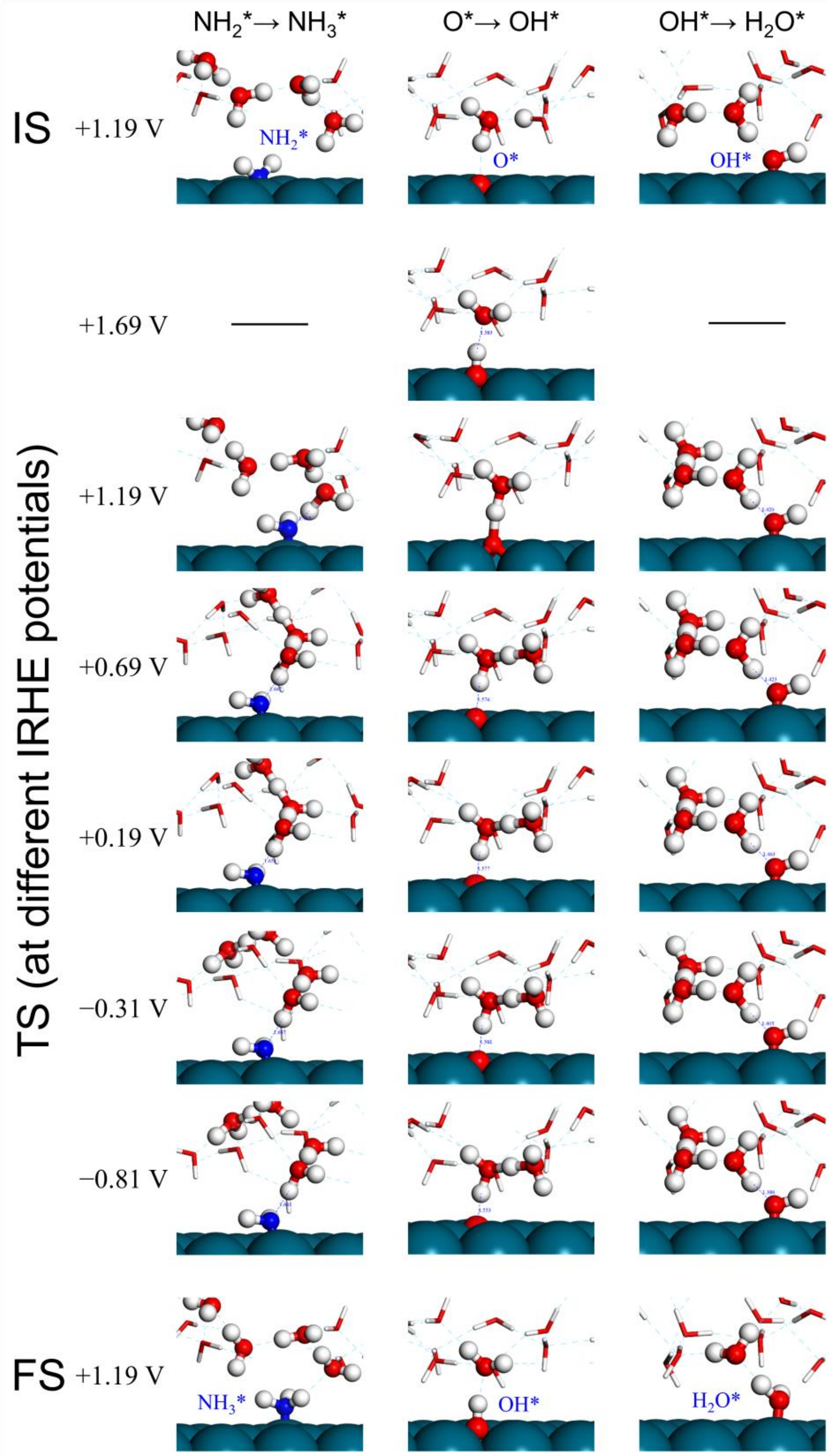

Fig. S5 Evolution of transition states for electrochemical steps: NH_2* + (H^+ + e^-) → NH_3*, O* + (H^+ + e^-) → OH* + H_2O and OH* + (H^+ + e^-) → H_2O*, with increased overpotentials, in acid conditions.

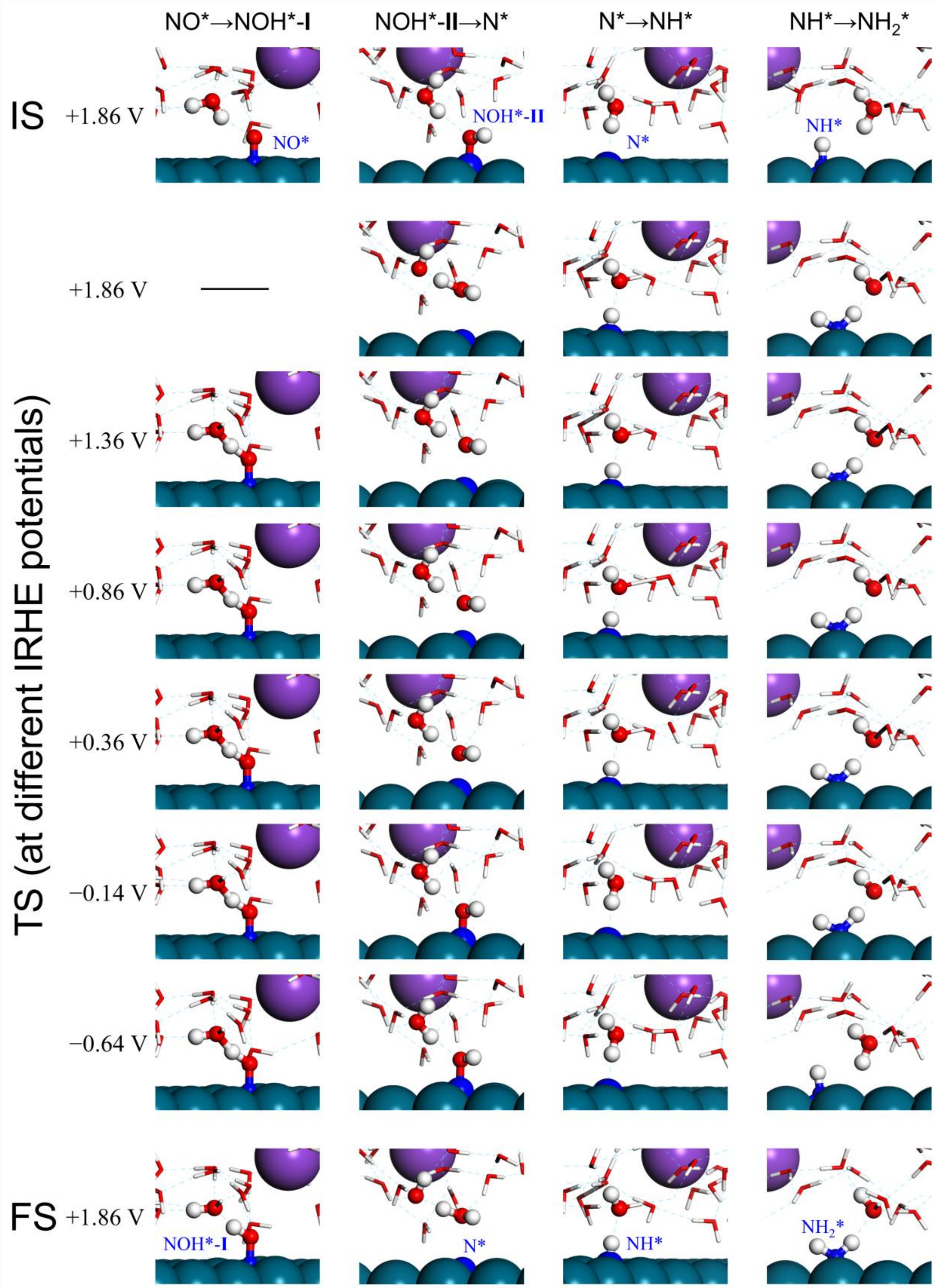

Fig. S6 Evolution of transition states for electrochemical steps: NO* + H_2O → NOH*-I + (OH^- − e^-), NOH*-II → N* + (OH^- − e^-), N* + H_2O → NH* + (OH^- − e^-) and NH* + H_2O → NH_2* + (OH^- − e^-), with increased overpotentials, in alkaline (neutral) conditions.

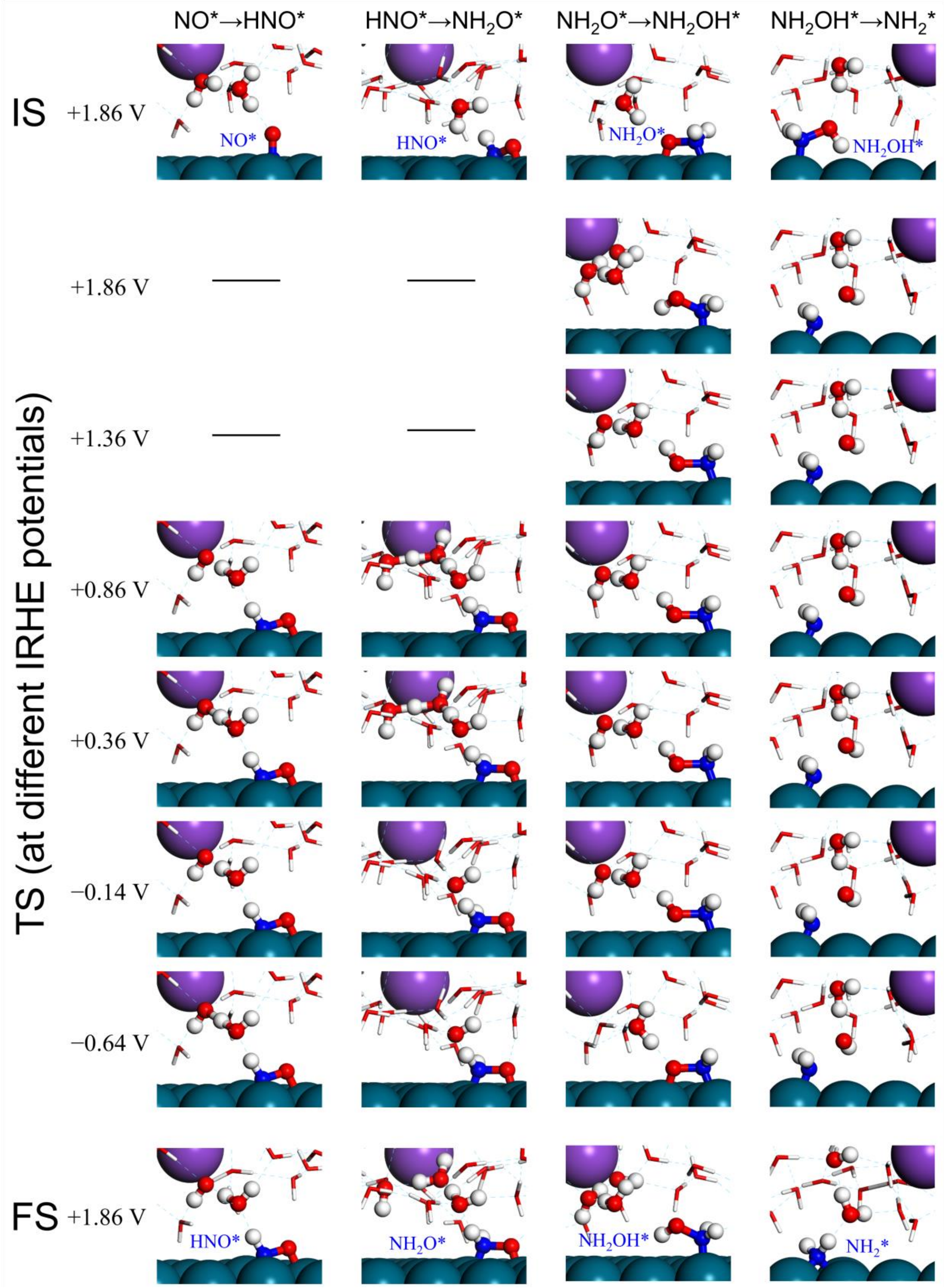

Fig. S7 Evolution of transition states for electrochemical steps: $NO^* + H_2O \rightarrow HNO^* + (OH^- - e^-)$, $HNO^* + H_2O \rightarrow NH_2O^* + (OH^- - e^-)$, $NH_2O^* + H_2O \rightarrow NH_2OH^* + (OH^- - e^-)$ and $NH_2OH^* \rightarrow NH_2^* + (OH^- - e^-)$, with increased overpotentials, in alkaline (neutral) conditions.

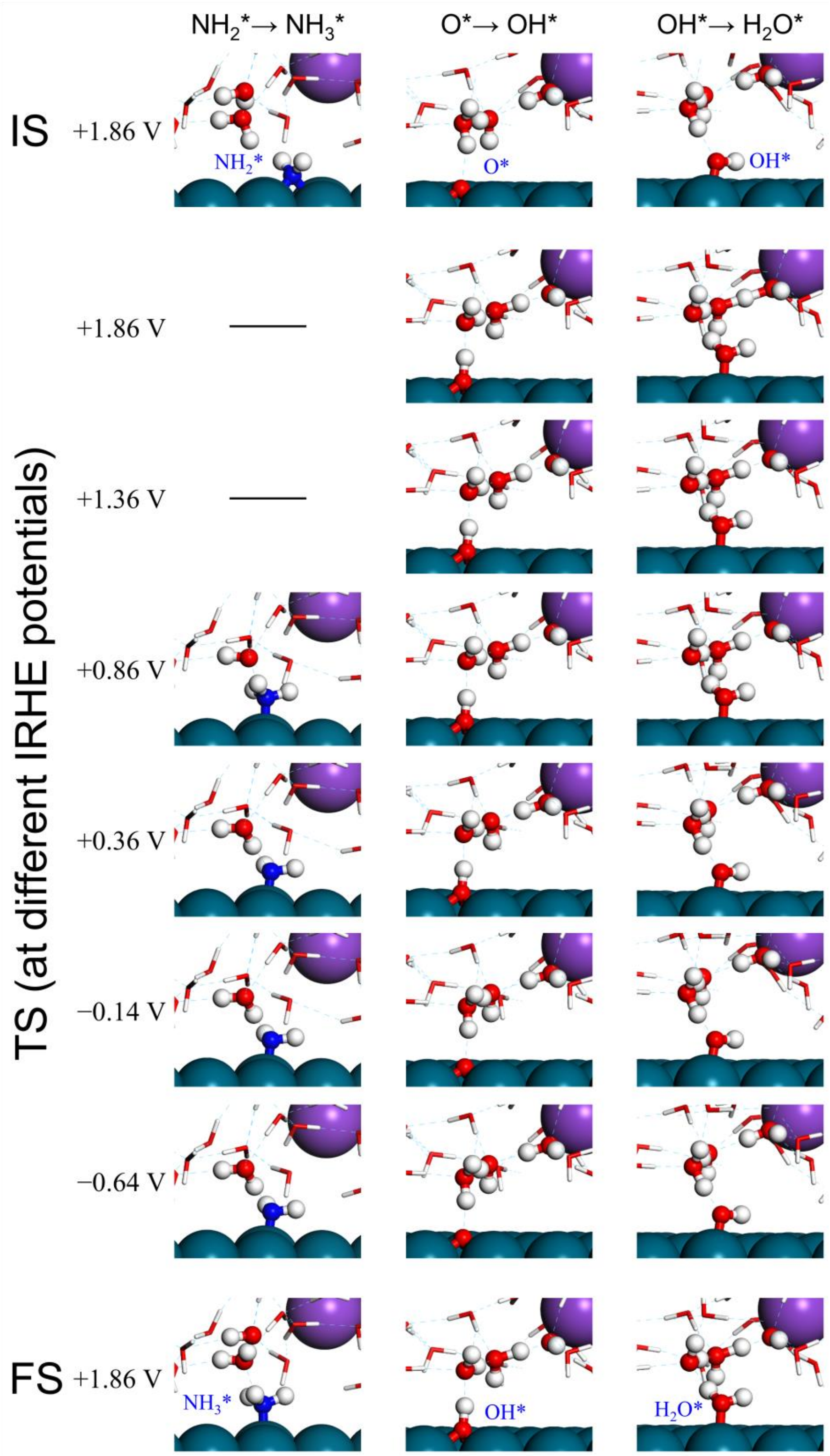

Fig. S8 Evolution of transition states for electrochemical steps: NH_2* + H_2O → NH_3* + (OH^- − e^-), O* + H_2O → OH* + (OH^- − e^-) and OH* + H_2O → H_2O* + (OH^- − e^-), with increased overpotentials, in alkaline (neutral) conditions.

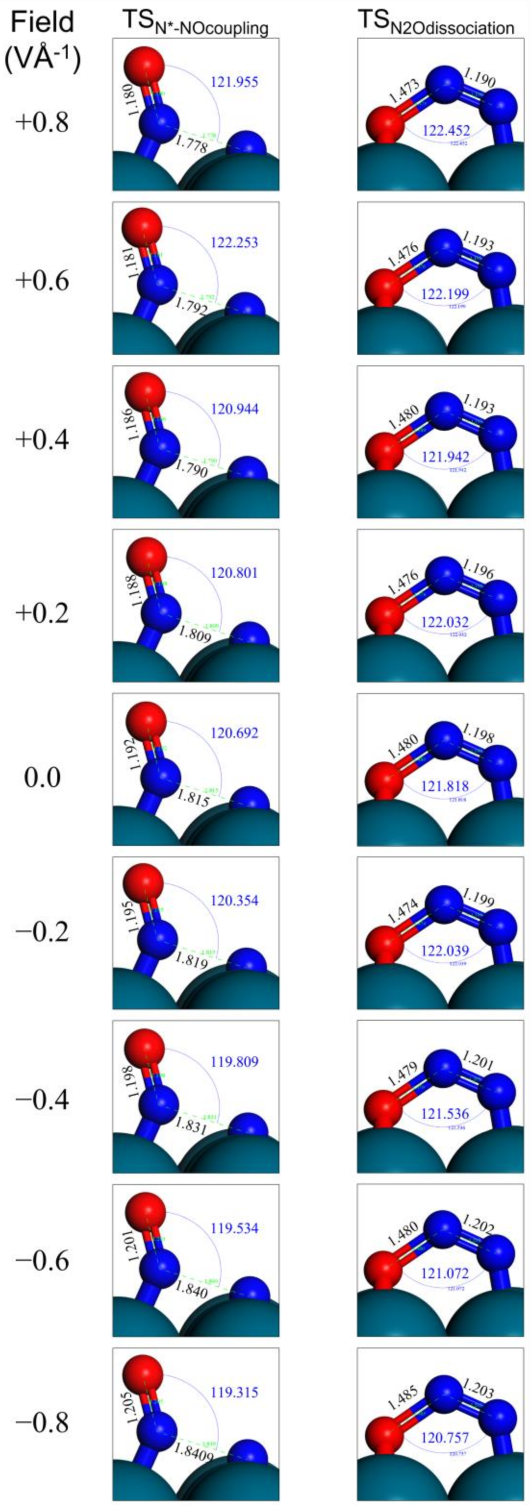

Fig. S9 Evolution of transition states for thermochemical steps (N*-NO coupling and N_2O dissociation) with varied electric field.

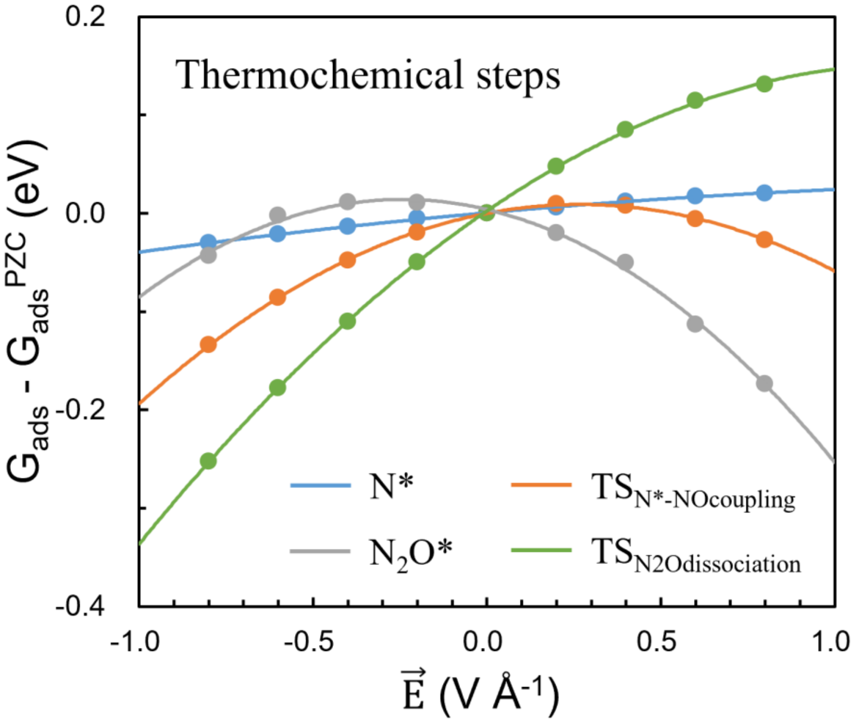

Fig. S10 Field effect on the relevant intermediates and transition states in thermochemical steps.

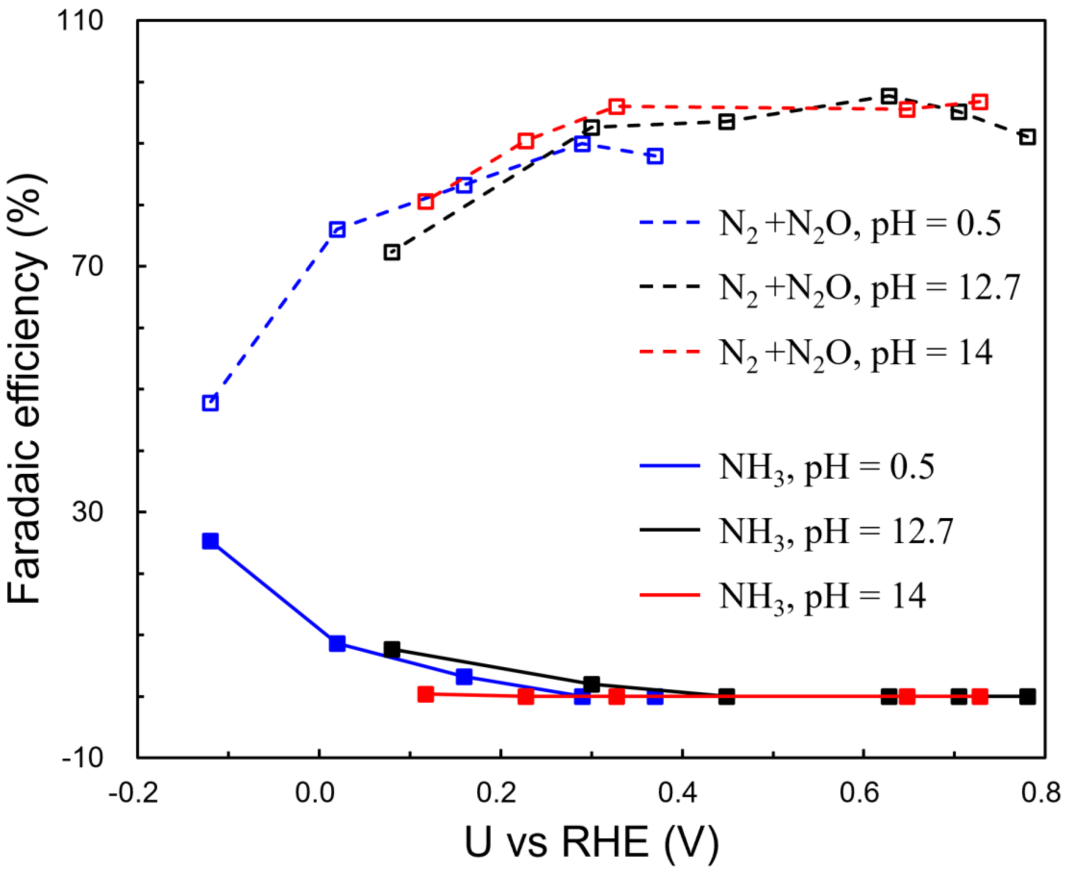

Fig. S11 Faradaic efficiency of dual-N products (N_2 and N_2O) and NH_3 at different pH[17].

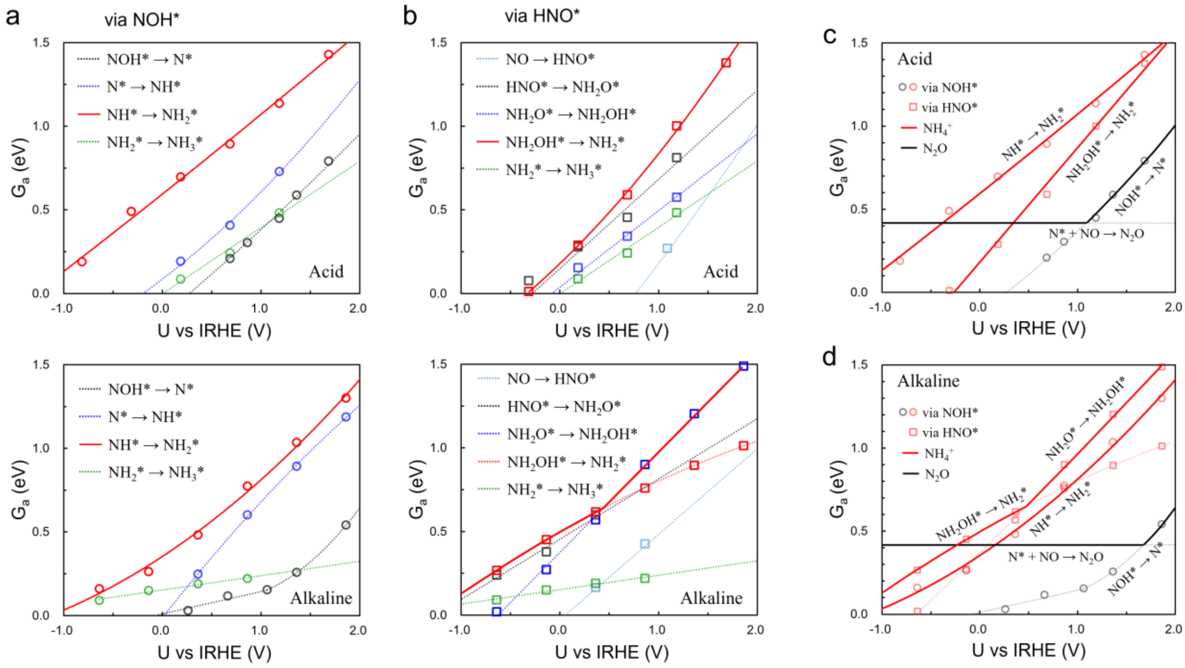

Fig. S12 Identification for the most energetically difficult steps (red solid lines) of NH_4^+ production via intermediates (**a**) NOH* and (**b**) HNO*. Selectivity analysis under (**c**) acid and (**d**) alkaline condition for NH_4^+ and N_2O productions, which are colored in red and black, respectively. The paths through intermediates NOH* and HNO* are presented by circle and square points, respectively, and the most energetically difficult steps of these paths are shown in solid lines.

Note:

As shown in **Fig. S12a**, for path via NOH* intermediates, NH* protonation to NH_2* has the highest barriers in both acid and alkaline conditions. When NH_4^+ yields through HNO* species, it not always has the same limiting steps at different pH (**Fig. S12b**). In acid solutions, the most difficult step is NH_2* formation from the dehydration of NH_2OH*. In alkaline conditions, the limiting steps are NH_2O* protonation and NH_2OH* dehydration, in other word, are NH_2* formation from the continuously protonation of NH_2O*. All in all, the barrier limiting step for NH_4^+ is the formation of NH_2*. As shown in **Fig. S12 c** and **d**, the barriers of NH_2* formation (red solid lines) are higher than that of the steps towards N_2O production (black solid lines) at low overpotentials, indicating the lower selectivity for NH_4^+ production. It is consistent with the experimental measurements (**Fig. S11**).

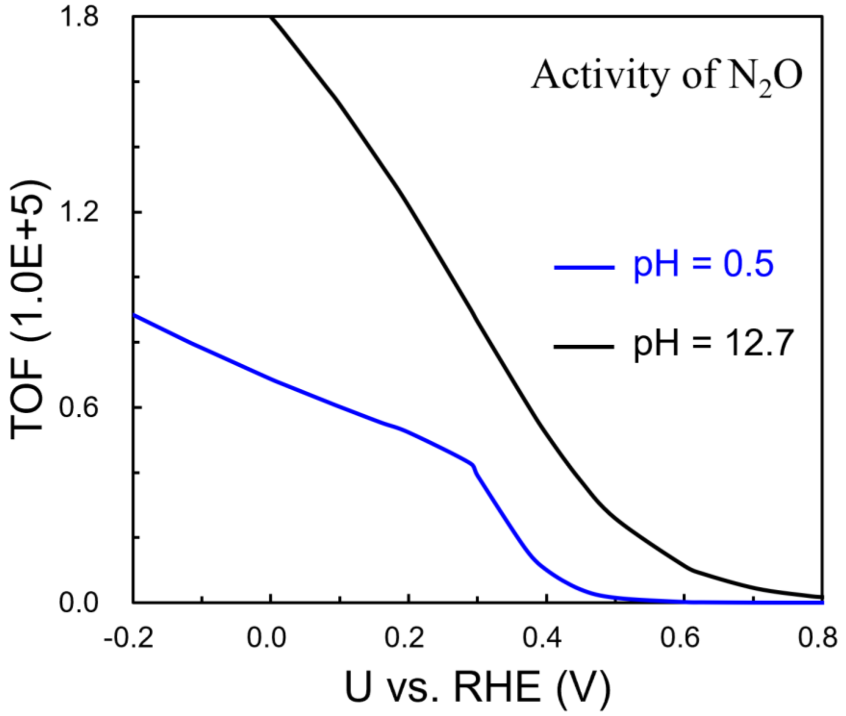

Fig. S13 Computational activity for N_2O production, simulated by pH-dependent microkinetic model.

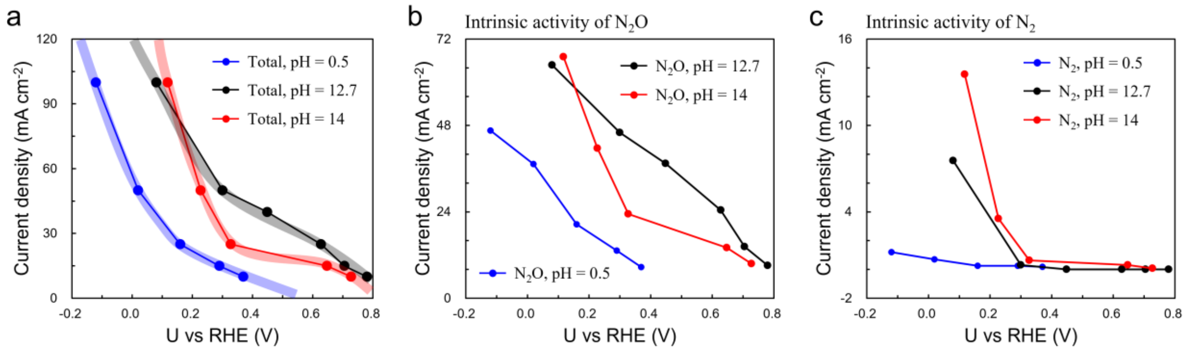

Fig. S14 (**a**) Total current density of eNORR and intrinsic partial current density for (**b**) N_2O and (**c**) N_2 products at pH = 0.5, 12.7 and 14.

Note:

In experiments, the partial current densities for products (j_i) are usually computed by their measured yields (n_i) and the total electron transfer number. As experiments proposed N_2 is produced directly from NO, an electron transfer number of 4 was used to computed the partial current densities of N_2 (j_{N2}). However, by considering the cascade mechanism (**Table S1**), that is, N_2 produced from N_2O, the total electron transfer number for N_2 production is 2. In other word, assuming all N_2 is produced from the secondary conversion of N_2O, the intrinsic current densities of N_2 ($j_{N2,int}$) should be a half of j_{N2} (Eq. S23).

$$j_{N_2,int} = \frac{1}{2} j_{N_2} \tag{S23}$$

Besides, the intrinsic yield of N_2O ($n_{N2O,int}$) should be higher than the measured value (n_{N2O}) in experiments, and $n_{N2O,int}$ equals the sum of n_{N2O} and n_{N2}. Note that the electron transfer number for N_2O production in eNORR is 4. Therefore, the intrinsic current densities of N_2O ($j_{N2O,int}$) can be calculated by

$$j_{N_2O,int} = j_{N_2O} + \frac{1}{2} j_{N_2} \tag{S24}$$

where j_{N2} and j_{N2O} refer to the partial current densities computed by proposing all N_2 is produced from NO.

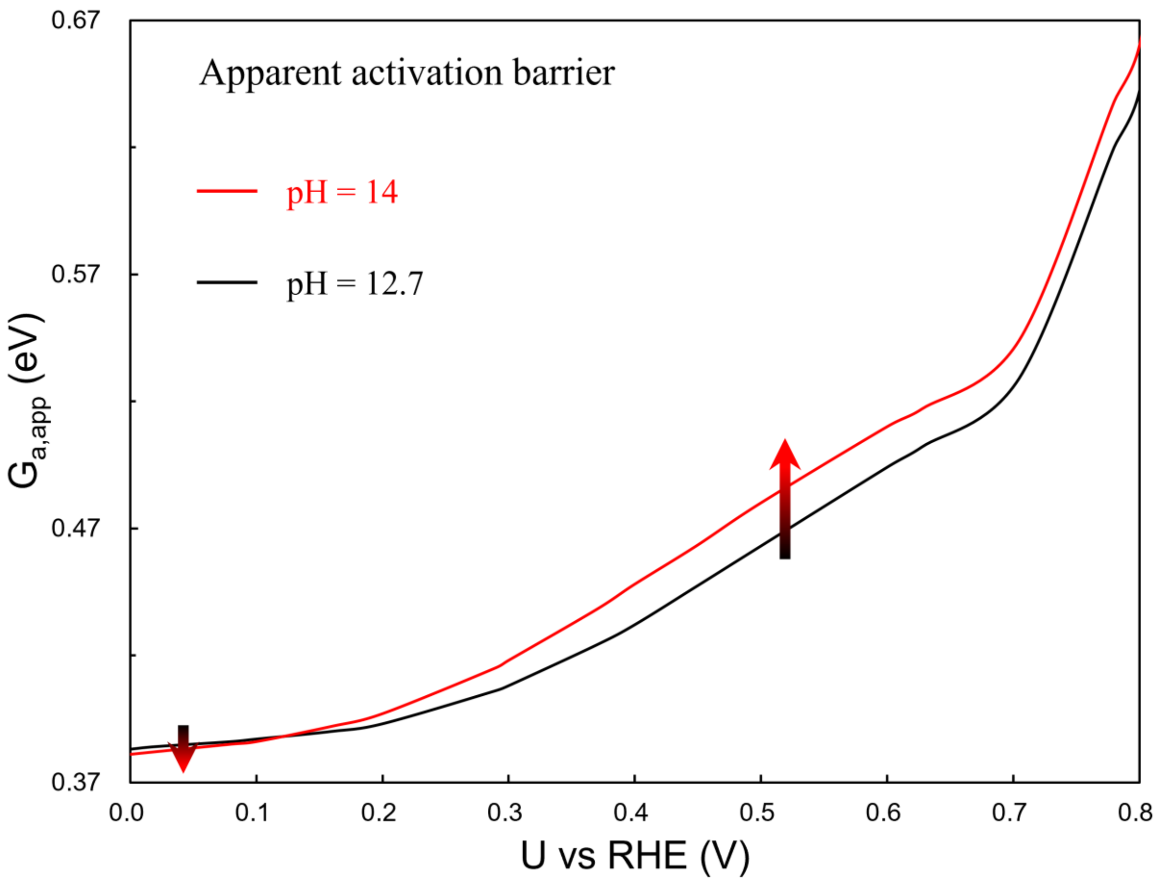

Fig. S15 Comparison of apparent activation barriers between pH = 12.7 and pH = 14.

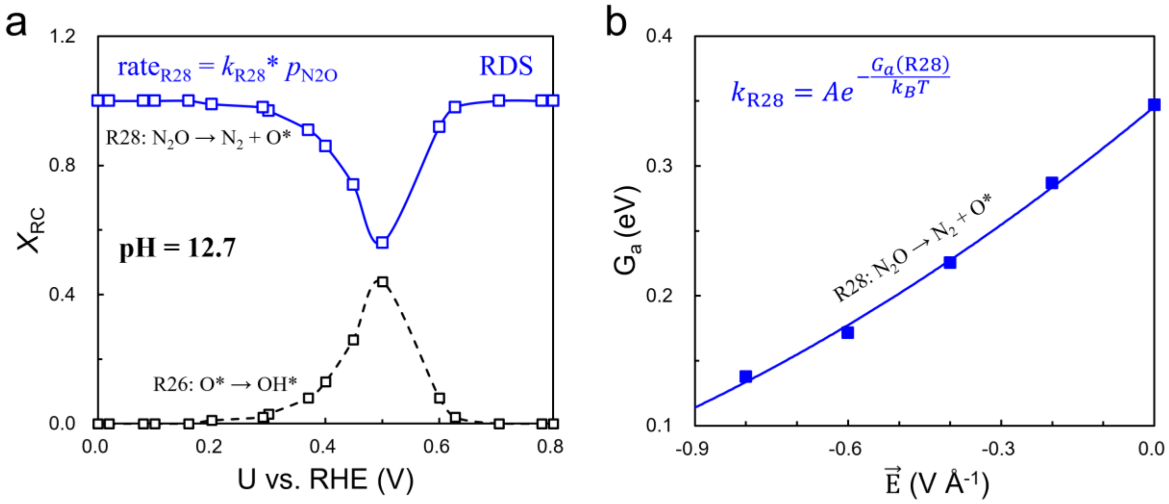

Fig. S16 (**a**) Degree of rate control (DRC) for different elementary steps (X_{RC}), simulated at pH = 12.7, towards N_2 production via cascade path. The rate-determining steps (RDS) are represented by blue solid line. (**b**) Relative low barriers for N_2O dissociation.

Note:

Considering cascade mechanism, N_2 yields by the secondary conversion of as-produced N_2O at electrochemical interface, where the partial pressure of N_2O (<0.01 atm) is extremely low due to the low conversion of NO. Besides, N_2 production has relatively low activation barrier too. As shown in **Fig. S16a**, N_2O dissociation limits the activity of N_2 production via cascade path, which has relatively low intrinsic barriers (**Fig. S16b**). Those two factors lead to the activity N_2 production more sensitive to partial pressure of N_2O than energetics.

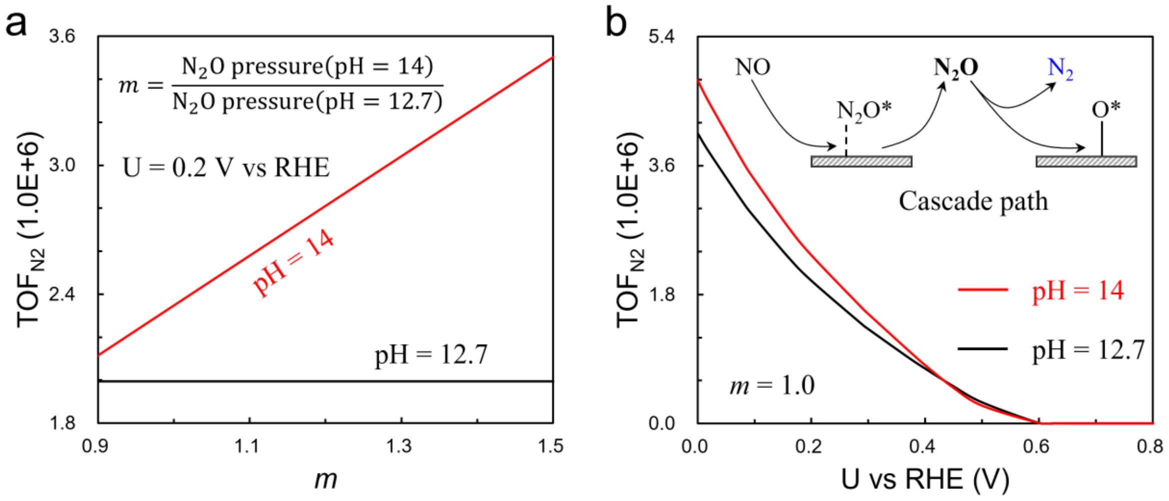

Fig. S17 (**a**) Comparison of N_2 activity between pH = 14 and 12.7, at 0.2 V vs RHE, where the partial pressure of reactant N_2O at pH = 14 is set to *m* times that at pH = 12.7. (**b**) Activity trend for N_2 production simulated with cascade mechanism, where the partial pressures of N_2O keep constant as pH increases from 12.7 to 14.

Note:

As the activity N_2 production is sensitive to partial pressure of N_2O, it was firstly tested to study the change in N_2 production rate as pH increases from 12.7 to 14. At pH = 12.7, we estimated N_2O pressure by the experimentally measured partial current densities (the intrinsic values in **Fig. S10**). At pH = 14, the partial pressures of N_2O were set to *m* times that at pH = 12.7. As shown in **Fig. S17a**, at 0.2 V vs RHE, the activity of N_2 at pH = 14 is constantly higher than that in pH = 12.7, in spite of some cases that N_2O pressure decreased slightly ($m < 1.0$). It originates from the slightly enhanced N_2O dissociation by more negative field (**Fig. S16b**). More importantly, the difference in N_2 activity between pH = 14 and pH = 12.7 obviously increases with *m* increasing (increased N_2O pressure at pH = 14). It indicates the high sensitivity of N_2O pressure on the N_2 production rate. Furthermore, at varying potentials, keeping N_2O pressure constant as pH increases from 12.7 to 14 (m = 1.0), **Fig. S17b** shows the consistent trends with experimental results (**Fig. 6c**). This confirms the rationality of cascade mechanism.

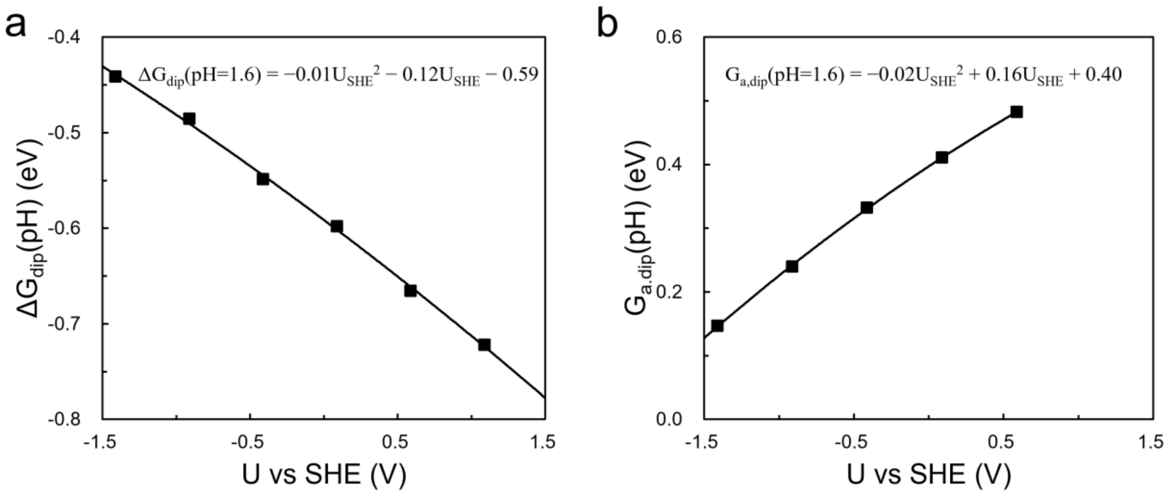

Fig. S18 Fitted functions of (**a**) $\Delta G_{dip}(pH=1.6)$ and (**b**) $G_{a,dip}(pH=1.6)$ against U_{SHE}, for exemplified step O* + ($H^+ + e^-$) → OH*.

Note:

1. $\Delta G_{dip}(pH=1.6)$ and $G_{a,dip}(pH=1.6)$ refer to the pH-dependent contributions (from adsorbate dipoles) to reaction free energy and barrier at pH = 1.6, respectively.

2. As potential (IRHE) decrease from 1.19 to 0.69 V (from 1.09 to 0.59 V vs SHE), the TS changes from a middle structure to IS-like structure, and it almost keeps intact when potential is lower than 0.69 V (**Fig. S5**). Thus, we only show the fitted line in the SHE potential region from 0.59 to −1.41 V, for $G_{a,dip}(pH=1.6)$. More calculations are needed to obtain this correlation when $U_{SHE} > 0.59$ V.

Table S1 The summary of considered pathways for NO electroreduction (eNORR).

**Acid**		**via NOH***		**via HNO***
NH_4^+	R1	$NO + (H^+ + e^-) \rightarrow NOH^*$	R13	$NO + (H^+ + e^-) \rightarrow HNO^*$
	R2	$NOH^* + (H^+ + e^-) \rightarrow N^* + H_2O$	R14	$HNO^* + (H^+ + e^-) \rightarrow NH_2O^*$
	R3	$N^* + (H^+ + e^-) \rightarrow NH^*$	R15	$NH_2O^* + (H^+ + e^-) \rightarrow NH_2OH^*$
	R4	$NH^* + (H^+ + e^-) \rightarrow NH_2^*$	R16	$NH_2OH^* + (H^+ + e^-) \rightarrow NH_2^* + H_2O$
	R5	$NH_2^* + (H^+ + e^-) \rightarrow NH_3^*$	R5	$NH_2^* + (H^+ + e^-) \rightarrow NH_3^*$
	R6	$NH_3^* + H^+ \rightarrow NH_4^+$	R6	$NH_3^* + H^+ \rightarrow NH_4^+$
N_2O	R1	$NO + (H^+ + e^-) \rightarrow NOH^*$		——————
	R2	$NOH^* + (H^+ + e^-) \rightarrow N^* + H_2O$		
	R7	$N^* + NO \rightarrow N_2O$		
N_2 (Sequential path)	R1	$NO + (H^+ + e^-) \rightarrow NOH^*$		——————
	R2	$NOH^* + (H^+ + e^-) \rightarrow N^* + H_2O$		
	R8	$N^* + NO \rightarrow N_2O^*$		
	R9	$N_2O^* \rightarrow N_2 + O^*$		
	R10	$O^* + (H^+ + e^-) \rightarrow OH^*$		
	R11	$OH^* + (H^+ + e^-) \rightarrow H_2O$		
N_2 (Cascade path)	R12	$N_2O \rightarrow N_2 + O^*$		——————
	R10	$O^* + (H^+ + e^-) \rightarrow OH^*$		
	R11	$OH^* + (H^+ + e^-) \rightarrow H_2O$		
**Alkaline (Neutral)**		**via NOH***		**via HNO***
NH_4^+	R17	$NO + H_2O \rightarrow NOH^* + (OH^- - e^-)$	R29	$NO + H_2O \rightarrow HNO^* + (OH^- - e^-)$
	R18	$NOH^* \rightarrow N^* + (OH^- - e^-)$	R30	$HNO^* + H_2O \rightarrow NH_2O^* + (OH^- - e^-)$
	R19	$N^* + H_2O \rightarrow NH^* + (OH^- - e^-)$	R31	$NH_2O^* + H_2O \rightarrow NH_2OH^* + (OH^- - e^-)$
	R20	$NH^* + H_2O \rightarrow NH_2^* + (OH^- - e^-)$	R32	$NH_2OH^* \rightarrow NH_2^* + (OH^- - e^-)$
	R21	$NH_2^* + H_2O \rightarrow NH_3^* + (OH^- - e^-)$	R21	$NH_2^* + H_2O \rightarrow NH_3^* + (OH^- - e^-)$
	R22	$NH_3^* + H_2O \rightarrow NH_3{\bullet}H_2O$	R22	$NH_3^* + H_2O \rightarrow NH_3{\bullet}H_2O$
N_2O	R17	$NO + H_2O \rightarrow NOH^* + (OH^- - e^-)$		——————
	R18	$NOH^* \rightarrow N^* + (OH^- - e^-)$		
	R23	$N^* + NO \rightarrow N_2O$		
N_2 (Sequential path)	R17	$NO + H_2O \rightarrow NOH^* + (OH^- - e^-)$		——————
	R18	$NOH^* \rightarrow N^* + (OH^- - e^-)$		
	R24	$N^* + NO \rightarrow N_2O^*$		
	R25	$N_2O^* \rightarrow N_2 + O^*$		
	R26	$O^* + H_2O \rightarrow OH^* + (OH^- - e^-)$		
	R27	$OH^* \rightarrow (OH^- - e^-)$		
N_2 (Cascade path)	R28	$N_2O \rightarrow N_2 + O^*$		——————
	R26	$O^* + H_2O \rightarrow OH^* + (OH^- - e^-)$		
	R27	$OH^* \rightarrow (OH^- - e^-)$		

Table S2 The adsorption free energy (in eV) of N_2O^* on various metal surfaces[15].

Surface	$G_{ad}N_2O^*$
Fe(110)	0.05
Co(111)	0.20
Ni(111)	0.14
Cu(111)	0.20
Pt(111)	0.28
Pd(111)	0.18
Au(111)	0.23
Ag(111)	0.25

References

(1) Kresse G and Furthmüller J. Efficiency of ab-initio total energy calculations for metals and semiconductors using a plane-wave basis set. *Comput Mater Sci* 1996; **6**: 15–50.

(2) Kresse G and Hafner J. Ab initio molecular-dynamics simulation of the liquid-metal–amorphous-semiconductor transition in germanium. *Phys Rev B* 1994; **49**: 14251–14269.

(3) Kresse G and Furthmüller J. Efficient iterative schemes for ab initio total-energy calculations using a plane-wave basis set. *Phys Rev B* 1996; **54**: 11169–11186.

(4) Hammer B, Hansen L and Nørskov J. Improved adsorption energetics within density-functional theory using revised Perdew-Burke-Ernzerhof functionals. *Phys Rev B* 1999; **59**: 7413–7421.

(5) Perdew J, Burke K and Ernzerhof M. Generalized gradient approximation made simple. *Phys Rev Lett* 1996; **77**: 3865–3868.

(6) Blöchl P, Jepsen O and Andersen O. Improved tetrahedron method for Brillouin-zone integrations. *Phys Rev B* 1994; **49**: 16223–16233.

(7) Kresse G and Joubert D. From ultrasoft pseudopotentials to the projector augmented-wave method. *Phys Rev B* 1999; **59**: 1758–1775.

(8) Cao X, Burch R and Hardacre C *et al*. An understanding of chemoselective hydrogenation on crotonaldehyde over Pt(111) in the free energy landscape: The microkinetics study based on first-principles calculations. *Catal Today* 2011; **165**: 71–79.

(9) Methfessel M and Paxton A. High-precision sampling for Brillouin-zone integration in metals. *Phys Rev B* 1989; **40**: 3616–3621.

(10) Luan D and Xiao J. Adaptive electric fields embedded electrochemical barrier calculations. *J Phys Chem Lett* 2023; **14**: 685−693.

(11) Borukhov I, Andelman D and Orland H. Adsorption of large ions from an electrolyte solution: a modified Poisson–Boltzmann equation. *Electrochim Acta* 2000; **46**: 221–229.

(12) Kelly S, Kirk C and Chan, K *et al*. Electric field effects in oxygen reduction kinetics: Rationalizing pH dependence at the Pt(111), Au(111), and Au(100) electrodes. *J Phys Chem C* 2020; **124**: 14581–14591.

(13) Nam K, Ishihara A and Matsuzawa K *et al*. Partially oxidized niobium carbonitride as a non-platinum catalyst for the reduction of oxygen in acidic medium. *Electrochim Acta* 2010; **55**: 7290–7297.

(14) Li H, Kelly S and Guevarra D *et al*. Analysis of the limitations in the oxygen reduction activity of transition metal oxide surfaces. *Nat Catal* 2021; **4**: 463–468.

(15) Li H, Long J and Jing H *et al*. Steering from electrochemical denitrification to ammonia synthesis. *Nat Commun* 2023; **14**: 112–122.

(16) Chen J, Jia M and Hu P *et al*. CATKINAS: A large-scale catalytic microkinetic analysis software for mechanism auto-analysis and catalyst screening. *J Comput Chem* 2021; **42**: 379–391.

(17) Ko B, Hasa B and Shin H *et al*. Electrochemical reduction of gaseous nitrogen oxides on transition metals at ambient conditions. *J Am Chem Soc* 2022; **144**: 1258–1266.
